# Evaluation of Concomitant Halogen and Pnictogen Bonds in Cocrystals of Imines Derived from 2-Nitrobenzaldehyde and 4-Haloaniline

**DOI:** 10.1021/acs.cgd.4c00102

**Published:** 2024-03-22

**Authors:** Nea Baus Topić, Nikola Bedeković, Leon Poljanić, Vladimir Stilinović, Dominik Cinčić

**Affiliations:** Department of Chemistry, Faculty of Science, University of Zagreb, Horvatovac 102a, Zagreb 10000, Croatia

## Abstract

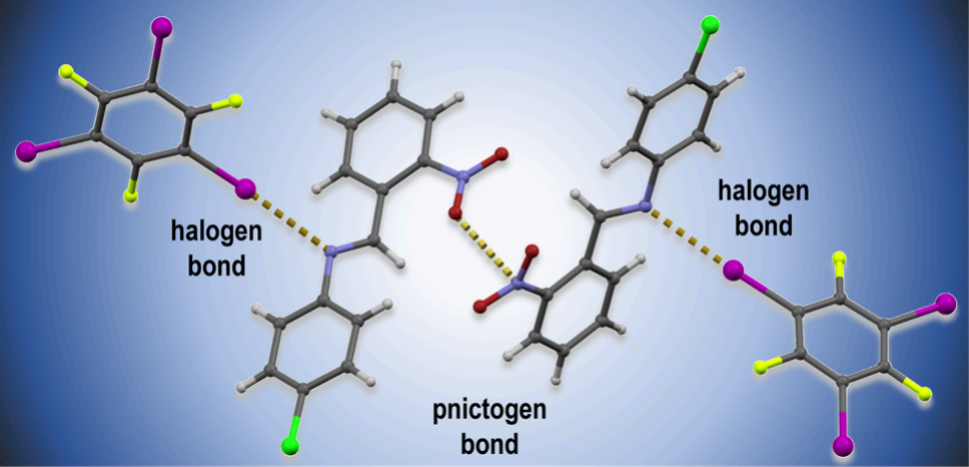

Three imines have been prepared by condensation of 2-nitrobenzaldehyde and 4-haloanilines (halo = Cl, Br, and I) with functionalities that enabled them to act as both halogen and pnictogen bond donors; however, both interactions were found to be absent in the solid state. The prepared imines were further cocrystallized with 1,3-diiodotetrafluorobenzene and 1,3,5-triiodotetrafluorobenzene as halogen bond donors. Six novel cocrystals were prepared by means of liquid-assisted mechanochemical synthesis and by crystallization from solution. All six cocrystals were of 1:1 stoichiometry and comprised a N···I halogen bond between an iodine atom of the perhalogenated halogen bond donor and the imino nitrogen atom of the imine acting as an acceptor. Additionally, in all six cocrystals, the imine molecules were interconnected by NO_2_···NO_2_ pnictogen bonding interactions. Computational analysis has shown that the NO_2_···NO_2_ exhibits bond critical point electron densities in the region (4.897–8.306) × 10^–3^ e Å^–3^ and interaction energies of 23.6–27.7 kJ mol^–1^, whereas the N···I halogen bonds generally have higher critical point electron densities ((1.795–1.937) × 10^–2^ e Å^–3^), but the corresponding total interaction energies are lower (19.4–20.4 kJ mol^–1^). Statistical analysis of the appearance of NO_2_···NO_2_ contacts concomitantly with halogen or hydrogen bonds seems to indicate that there is a positive correlation between the presence of NO_2_···NO_2_ pnictogen bonding interactions and other directional interactions in crystal structures.

## Abstract 2

Imines derived from 2-nitrobenzaldehyde and 4-haloanilines (halo = Cl, Br, and I) have been cocrystallized with two halogen bond donors. In the obtained cocrystals, a halogen bond was formed with the imine nitrogen atom, while nitro groups of neighboring imine molecules formed NO_2_···NO_2_ pnictogen bonding contacts.

## Introduction

Over the past three decades, the study of halogen bonding became one of the most consistently developing areas in supramolecular chemistry and crystal engineering,^1^ mainly because of its usefulness in the design of crystal materials with desired properties and supramolecular topologies.^1−5^

While most commonly used in the context of crystal engineering to date, halogen and hydrogen bonds are not the only noncovalent interactions that arise from the anisotropic distribution of electronic density around donor and acceptor atoms, which creates areas of lower electronic density in the continuation of the covalent bond (σ-hole) or perpendicular to a system of coplanar bonds (π-hole), leading to areas of positive electrostatic potential enabling the atoms to act as Lewis acids (electrophiles). Such electron anisotropy can occur in covalently bound atoms of many elements, and the resulting interactions are usually classified according to the group of the periodic table of elements to which the atom acting as a Lewis acid belongs.^6^ Thus, along the halogen bond (Group 17), one can speak of chalcogen (Group 16),^7^ pnictogen (Group 15),^8^ tetrel (Group 14),^9−11^ triel (Group 13),^12^ and noble gas or aerogen bonds (Group 18),^13^ as well as the metal-centered regium (Group 11)^14^ and spodium bond (Group 12).^15,16^

Pnictogen bonding has been found in numerous molecular solids comprising pnictogen atoms but has not yet found its application in crystal engineering to the same extent as hydrogen or halogen bonding.^17^ Compared to the halogen bond, the pnictogen bond is less directional, which is a result of total steric and electronic asymmetry around pnictogen atoms.^18^ Moreover, deviation from the linearity can be explained in the repulsive interactions of pnictogen-bonded nucleophiles with lone electron pairs of the pnictogen and the atoms (covalently) bound to the pnictogen atom.^19,20^ Nevertheless, the pnictogen bond is a noteworthy supramolecular interaction, which in some cases (such as P···P pnictogen bonds), can exhibit bond strength comparable to that of the hydrogen bond.^21^ Pnictogen bonding has been used in the design of functionalized materials for anion binding,^22^ noncovalent catalysis,^23^ and molecular recognition.^24^ One of the first functional groups found to participate in pnictogen bonding is a nitro group, the nitrogen atom of which can act as a π-hole donor.^25,27,28^ The π-hole of the nitro group has been found to form directional bonds with electron-rich atoms,^26−29^ such as oxygen atoms from a neighboring nitro group, which was demonstrated to be an attractive interaction by Woźniak et al. in 1994.^30^ Further studies have shown that NO_2_···NO_2_ have similar energies as C–H···O weak hydrogen bonds^31^ and are a common feature in crystal structures.^32^ Although most of the earlier studies have concentrated on NO_2_···NO_2_ interactions where the two nitro groups are in approximate perpendicular arrangement (allowing for a close approach of an oxygen atom of one nitro group to the nitrogen atom of the other), more recent studies seem to suggest that the NO_2_···NO_2_ interaction is more favorable when the planes of the NO_2_ groups are parallel.^33^ A recent statistical analysis and theoretical study on NO_2_···NO_2_ interactions between nitrobenzene moieties by Saha et al. has demonstrated that parallel arrangement of interacting NO_2_ groups in nitrobenzene molecules is not only energetically more favorable (−22.05 kJ mol^–1^ for nitrobenzene) than the perpendicular arrangement (−18.16 kJ mol^–1^) but is also more common among the crystal structures of aromatic nitro compounds.^34^

While the NO_2_···NO_2_ interactions have been extensively studied, there has been little work done on systems in which they coexist with other interactions. The simplest way to achieve this is to study molecules with multiple functionalities capable of forming different intermolecular interactions. Convenient molecular scaffolds are imines, relatively easily prepared by condensation reaction of amines (mostly primary) and carbonyl compounds that allow for simple combination of two fragments that can carry different functionalities.^35^ Traditionally, imines have been studied as ligands in coordination compounds,^36^ as model systems for study of tautomerism,^37−40^ biological macromolecules,^41^ synthetic intermediates,^42^ and anion receptors.^43^ More recently, however, imines and their coordination compounds also started drawing attention in crystal engineering, particularly halogen-bonded cocrystals.^44−46^ Mostly, this includes functionalizing the imine with halogen bond acceptor groups, such as a piperidinyl^45,47,48^ or nitrile^44^ nitrogen atom or a carbonyl,^44,49^ methoxy,^44,46,48^ hydroxyl,^44−48^ morpholinyl,^49,50^ or nitro^51^ oxygen atom, although there are few studies where appropriately functionalized imines have been used as halogen bond donors.^52,53^ Thus, a combination of a nitro group and halogen atoms in the same imine could afford data on the interrelationship between NO_2_···NO_2_ interactions and a halogen bond. To the best of our knowledge, only one systematic study of a series of such imines combining nitro groups and iodine has been published to date.^54,55^ Within the studied series of compounds (nine nitrobenzylideneiodoaniline isomers, some appearing in several polymorphic forms), the I···O_nitro_ halogen bond was observed in three and the I···N_imine_ halogen bond in one structure, while the NO_2_···NO_2_ interactions were also found in only one structure.

**Scheme 1:**
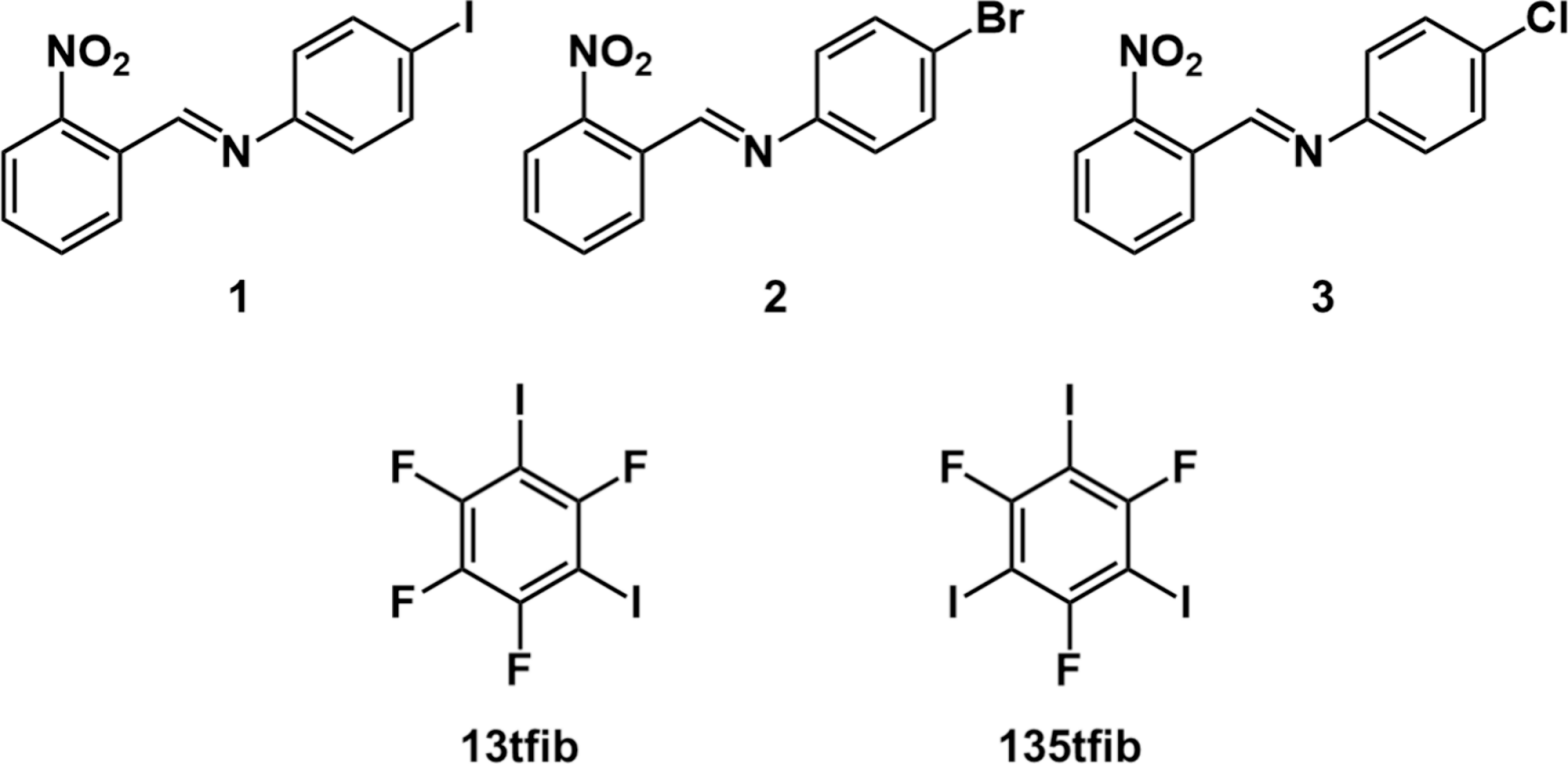
Molecular Diagrams of the Imines (**1**, **2**, and **3**) and the Halogen Bond Donors (**13tfib** and **135tfib**) Used in the Study

For the current study, we have also selected imines functionalized with both a halogen atom (potential halogen bond donor) and a nitro group, thus preparing a series of three 2-nitrobenzylidene-4′-haloanilines (halo = iodo, bromo, and chloro; Scheme 1). However, to increase the probability of a halogen bond occurring in the structure, the imines were also cocrystallized with two halogen bond donors (1,3-diiodoterafluorobenzene, **13tfib**, and 1,3,5-triiodotrifluorobenzene, **135tfib**). This should enable us to observe potential competition of the binding sites on the imine molecule (oxygen atoms of the nitro group and nitrogen atom of the imine group) for the halogen atoms as well as possible occurrence of concomitant NO_2_···NO_2_ pnictogen bonds.

## Results and Discussion

**Figure 1:**
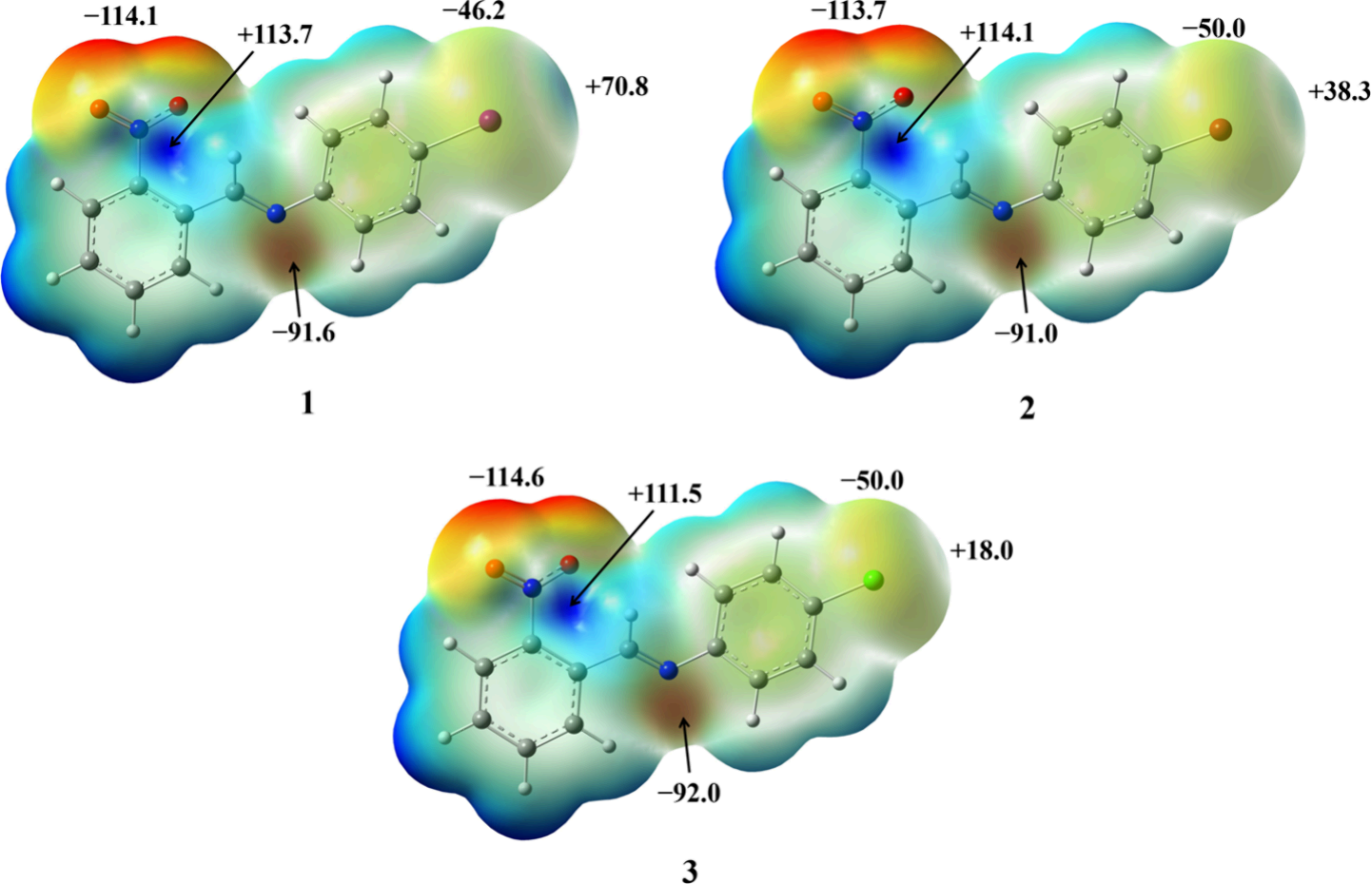
Optimized geometries and molecular electrostatic potential mapped on the total electron density isosurface (1.0 × 10^–3^ e A^–3^) of **1**, **2**, and **3**. All values are given in kJ mol^–1^ e^–1^.

Given that the selected imine molecules contain several functional groups able to participate in halogen bonding, we have performed DFT calculations of molecular electrostatic potential (MEP) to characterize their donor–acceptor potential. It can be noticed (Figure 1) that in all three molecules, the most negative MEP values are found on the oxygen atoms of the nitro group (−113.7 to −114.6 kJ mol^–1^ e^–1^), which are followed by the imine nitrogen atoms (−91.0 to −92.0 kJ mol^–1^ e^–1^). The area of the most positive electrostatic potential was found in the immediate vicinity of the nitrogen atom of the nitro group (+111.5 to +114.1 kJ mol^–1^ e^–1^), while the σ-holes on the halogen atoms are considerably less positive (ranging from +18.0 kJ mol^–1^ e^–1^ in **3** to +70.8 kJ mol^–1^ e^–1^ in **1**) compared with the π-holes on the nitro groups. According to the given data, participation of Schiff base molecules in halogen bonding with iodine atom(s) from perfluorinated donor molecules can be expected either via nitro oxygen or imine nitrogen atoms as well as participation in NO_2_···NO_2_ contacts. However, the formation of halogen bonds in which imine molecules act as halogen bond donors seems to be unlikely (except possibly in **1**) due to the relatively small MEP values of the σ-holes of halogen atoms.

**Figure 2:**
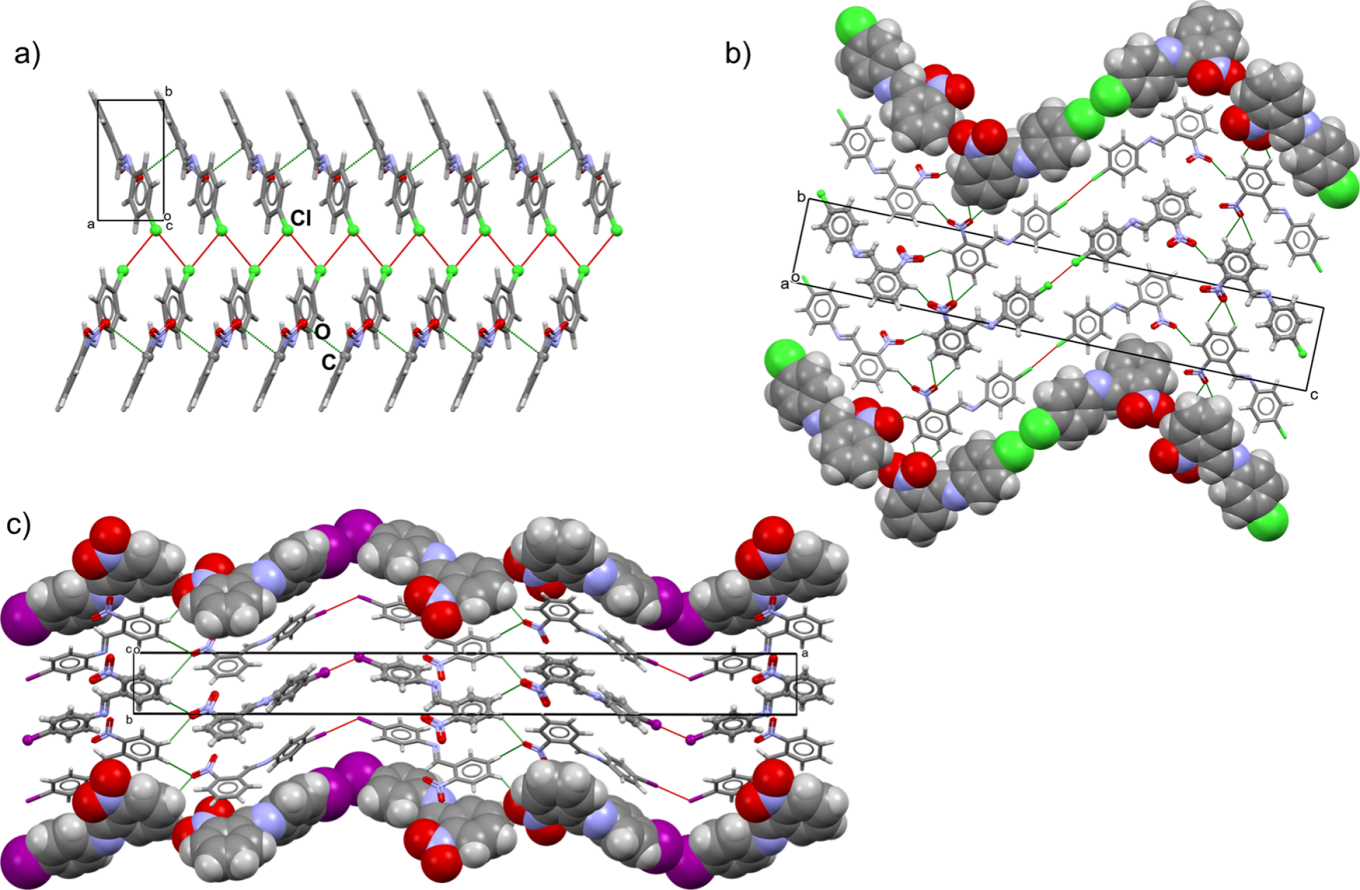
(a) Supramolecular chains along the *a* axis in **3** achieved through Cl···Cl interhalogen contacts. (b) Interconnection of chains via C–H···O_nitro_ hydrogen bonds into the 3D network in **3** viewed along the *a* axis. Analogous chains and networks are formed in **2** (Figure S42). (c) 3D network in **1** (XUDYAW) viewed along the *c* axis. Interhalogen interactions are shown as red dotted lines, while the other interactions are shown as green dotted lines.

All three targeted imines have been prepared by synthesis from solution (a detailed experimental description can be found in the SI), and single crystals suitable for X-ray diffraction experiments have been obtained. Of the three, the crystal structure of **1** has been previously reported.^54^ Two novel imines, **2** and **3**, are isostructural (unit cell similarity index (π) of 0.0231 and isostructurality index (*I*_s_) of 77.23%) but are not isostructural with **1**. In the crystal structures of **2** and **3**, the imine molecules are connected by type II interhalogen contacts (*d*_rel_(Br···Br) = 82.0%, *d*_rel_(Cl···Cl) = 85.4%) and also form C(π)···O_nitro_ short contacts (**2***d*_rel_(C···O) = 86.2% and **3***d*_rel_(C···O) = 86.4%), which result in the formation of supramolecular chains extended along the *a* axis (Figure 2a, Figure S42a).^56^ The chains are interconnected by additional C–H···O_nitro_ hydrogen bonds into a 3D network (Figure 2b, Figure S42b). Both oxygen atoms from the nitro group are involved in hydrogen bonding; one is bifurcated, and the other is only a single hydrogen bond acceptor. In the structure of **1**, there are two symmetrically inequivalent molecules in the asymmetric unit that are connected via type II interhalogen interaction (*d*_rel_(I···I) = 79.0%). The supramolecular chains are formed through altering I···I contacts and C–H···O_nitro_ hydrogen bonds and are stacked into a 3D network by additional C–H···O_nitro_ interactions (Figure 2c).

Interestingly, in spite of the high electrostatic potential of the NO_2_ nitrogen atom of the nitro group, in neither structure does the nitro group act as a pnictogen bond donor. Less surprisingly (taken into account the low values of electrostatic potential corresponding to the σ-holes of the halogen atoms), the halogen bonds involving the nitro group were also found to be absent. To attempt to introduce a halogen bond into the system, we prepared cocrystals of the three selected imines with stronger halogen bond donors (**13tfib** and **135tfib**). Six cocrystals (**1**)(**13tfib**), (**1**)(**135tfib**), (**2**)(**13tfib**), (**2**)(**135tfib**), (**3**)(**13tfib**), and (**3**)(**135tfib**) were prepared mechanochemically. Identical phases have also been prepared by cocrystallization from solution, which resulted in formation of single crystals suitable for X-ray diffraction experiments.

**Figure 3:**
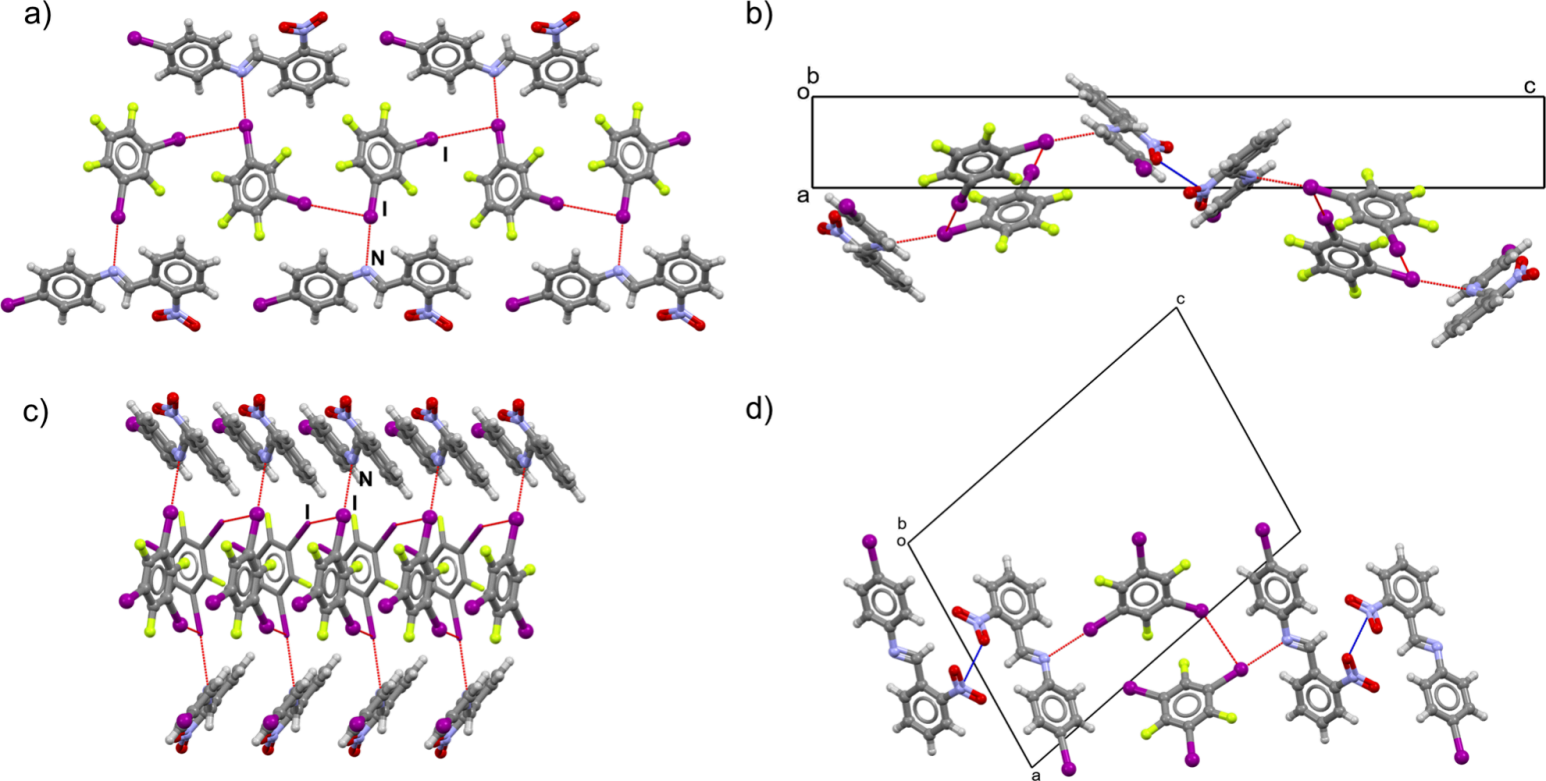
Halogen-bonded chains in (a) (**1**)(**13tfib**) and (c) (**1**)(**135tfib**). Pnictogen bonds between imine molecules of neighboring halogen-bonded chains in (b) (**1**)(**13tfib**) and (d) (**1**)(**135tfib**).

In both cocrystals of **1**, the halogen bonding occurs between an iodine atom of the donor molecule and the imine nitrogen of **1**, and they are of almost identical lengths (ca. 2.97 Å), although the halogen bond in (**1**)(**135tfib**) is more linear (θ(C–I···N) = 177.5(2)°) than the one found in (**1**)(**13tfib**) (θ(C–I···N) = 172.8(6)°; Table 1). The second iodine of the donor molecules participates in type II interhalogen interaction with the iodine atom of the adjacent donor molecule, which leads to the formation of halogen-bonded supramolecular chains of **13tfib** (Figure 3a) or **135tfib** (Figure 3c) molecules extended along the *b* axis. The chains are decorated with halogen-bonded imine molecules alternatively on either side of the chain. In cocrystal (**1**)(**135tfib**), the third iodine atom of the donor participates in interactions with fluorine atoms of neighboring chains, which lead to the formation of a 3D network (Figure S43). The iodine atom of molecule **1** does not participate in halogen bonding in any significant way. In both cocrystals, two neighboring molecules of **1** are also connected by NO_2_···NO_2_ pnictogen bonds (Figure 3b,d; Table 2) of 3.263(7) and 3.13(2) Å, respectively.

**Table 1:** Lengths (*d*), Relative Lengths (*d*_rel_), Angles (θ), and Electron Densities at the Bond Critical Points (ρ_bcp_) of Halogen Bonds in the Crystal Structures of Cocrystals of **1**, **2**, and **3** with **13tfib** and **135tfib**

cocrystal	halogen bond with symmetry code	*d*/Å	*d*_rel_^a^/%	θ_1_(C–I···N)/°	θ_2_(C–I···I)^b^/°	ρ_bcp_*/* e Å^–3^
(**1**)(**13tfib**)	I2···N1	2.97(1)	71.2	172.8(6)		1.881 × 10^–2^
	*x*, *y*, *z*					
	I2···I3	3.951(2)	83.0	168.1(5)	81.6(5)	
	2 – *x*, 1/2 + *y*, 1/2 – *z*					
(**1**)(**135tfib**)	I2···N1	2.975(6)	71.3	177.5(2)		1.902 × 10^–2^
	*x*, *y*, *z*					
	I2···I4	4.0385(8)	84.8	143.7(2)	84.7(2)	
	2 – *x*, −1/2 + *y*, 1 – *z*					
(**2**)(**13tfib**)	I1···N1	2.963(7)	71.1	172.3(2)		1.937 × 10^–2^
	*x*, *y*, *z*					
	I1···I2	3.8385(8)	80.6	167.7(2)	80.5(2)	
	*–x, −*1/2 + *y*, 3/2 *– z*					
(**2**)(**135tfib**)	I1···N1	2.973(4)	71.3	177.1(2)		1.907 × 10^–2^
	*x*, *y*, *z*					
	I1···I3	3.9460(6)	82.9	144.2(2)	83.7(1)	
	2 – *x*, −1/2 + *y*, 1 – *z*					
(**3**)(**13tfib**)	I1···N1	3.001(3)	72.0	173.1(1)		1.795 × 10^–2^
	*x*, *y*, *z*					
(**3**)(**135tfib**)	I1···N1	2.967(8)	71.2	172.2(3)		1.936 × 10^–2^
	*x*, *y*, *z*					
	I1···I2	3.9563(9)	83.1	146.8(3)	80.5(2)	
	1/2 – *x*, −1/2 + *y*, 3/2 – *z*					

a *d*_rel_ = 100 × [*d*//(*r*_vdW_(I) + *r*_vdW_(*N/I*))].^56^

b For type II interhalogen interactions.

**Table 2:** Lengths (*d*), Relative Lengths (*d*_rel_), Angles (θ), and Electron Densities at the Bond Critical Points (ρ_bcp_) of Pnictogen Bonds in the Crystal Structures of Cocrystals of **1**, **2**, and **3** with **13tfib** and **135tfib**

cocrystal	pnictogen bond with symmetry code	*d*/Å	*d*_rel_^a^/%	θ(N–O···N)/°	ρ_bcp_*/* e Å^–3^
(**1**)(**13tfib**)	N2···O2	3.13(2)	89.4	138(1)	5.677 × 10^–3^
	1/2 + *x*, 3/2 – *y*, 1 – *z*				
(**1**)(**135tfib**)	N2···O2	3.263(9)	93.2	141.4(5)	4.897 × 10^–3^
	1 – *x*, −1/2 + *y*, −*z*				
(**2**)(**13tfib**)	N2···O1	3.08(1)	88.0	136.5(5)	5.695 × 10^–3^
	–1/2 + *x*, 1/2 – *y*, 1 – *z*				
(**2**)(**135tfib**)	N2···O2	3.077(7)	87.9	138.8(4)	5.876 × 10^–3^
	1 – *x*, −1/2 + *y*, −*z*				
(**3**)(**13tfib**)	N2···O1	3.115(5)	89.0	134.8(3)	5.666 × 10^–3^
	1 – *x*, −1/2 + *y*, 3/2 – *z*				
(**3**)(**135tfib**)	N2···O1	2.88(1)	82.3	133.9(6)	8.306 × 10^–3^
	3/2 – *x*, −1/2 + *y*, 3/2 – *z*				

a *d*_rel_ = 100 × [*d*//(*r*_vdW_(N) + *r*_vdW_(O))].^56^

**Figure 4:**
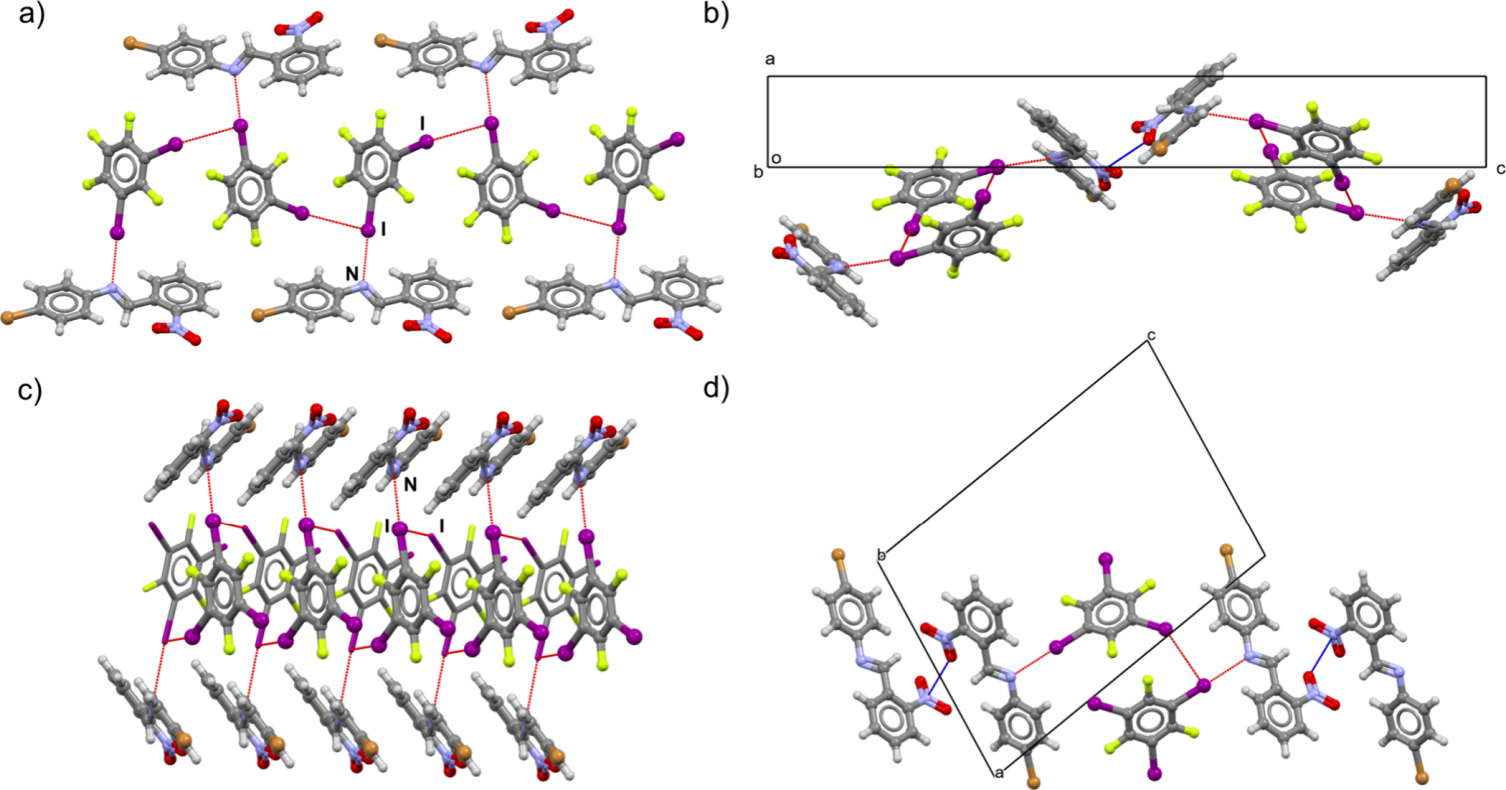
Halogen-bonded chains in (a) (**2**)(**13tfib**) and (c) (**2**)(**135tfib**). Pnictogen bonds between imine molecules of neighboring halogen-bonded chains in (b) (**2**)(**13tfib**) and (d) (**2**)(**135tfib**).

The two cocrystals derived from **2**, (**2**)(**135tfib**) and (**2**)(**13tfib**), are isostructural with their **1** analogues. The unit cell similarity index (π) for cocrystals with **13tfib** is 0.0065, and the isostructurality index (*I*_s_) is 85.2%, while π for cocrystals with **135tfib** is 0.0123 and *I*_s_ 79.6%. Following the isostructurality of cocrystals, supramolecular interactions and motifs in (**2**)(**13tfib**) and (**2**)(**135tfib**) correspond to those found in cocrystals of **1** (Figure 4). Halogen bond geometries are quite similar in halogen-bonded cocrystals of **2** and **1** (Table 1), while N···O distances in cocrystals of **1** are generally somewhat longer than in cocrystals of **2** (Table 2), which can be attributed to the reduction of the size of the halogen substituent on the imine (iodine in **1** vs bromine in **2**), which also reduces the distances between molecules of **2** in neighboring supramolecular layers.

**Figure 5:**
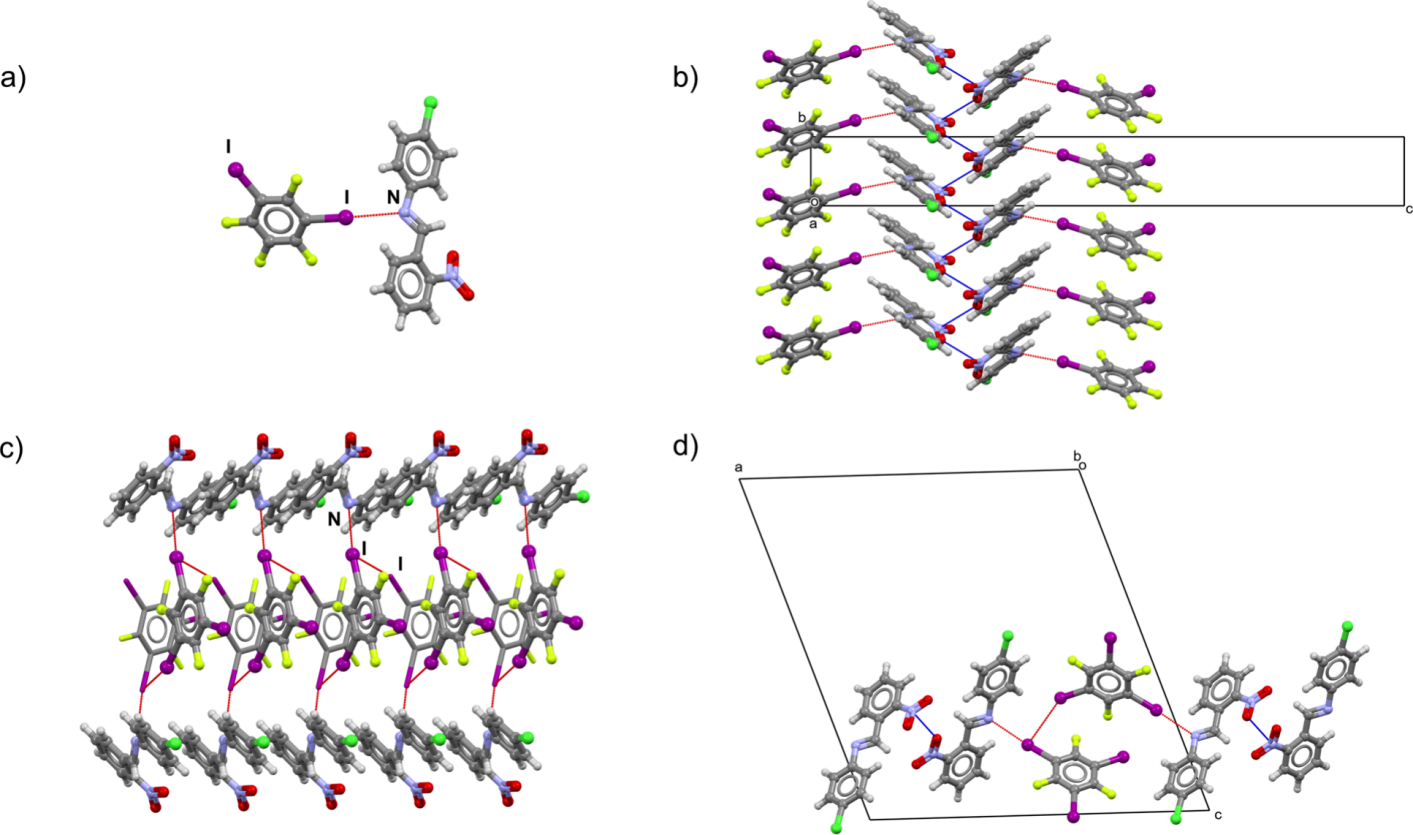
(a) Discrete halogen-bonded dimers in (**3**)(**13tfib**). (b) Pnictogen-bonded chains in (**3**)(**13tfib**). (c) Halogen-bonded chains in (**3**)(**135tfib**). (d) Pnictogen bonds between **3** molecules of neighboring halogen-bonded chains in (**3**)(**135tfib**).

Although **3** is isostructrural with **2**, the cocrystals that the two imines form with **13tfib** and **135tfib** are not. In (**3**)(**13tfib**), donor and acceptor molecules form halogen-bonded dimers via I···N_imine_ halogen bonds (Figure 5a). This is the only cocrystal in which halogen-bonded supramolecular chains are not formed, and no type II interhalogen interaction is established. The dimers are interconnected by NO_2_···NO_2_ pnictogen bonds, which leads to formation of pnictogen-bonded zigzag chains extended along the *b* axis (Figure 5b). In (**3**)(**135tfib**), the supramolecular motifs formed through halogen (Figure 5c) and pnictogen bonding (Figure 5d) are analogous to those in (**1**)(**135tfib**) and (**2**)(**135tfib**). Halogen bond lengths and angles in cocrystals with **3** are comparable to those found in cocrystals of **2** and **1**. In both (**1**)(**135tfib**) and (**2**)(**135tfib**), two adjacent imine molecules participate in C–H···O hydrogen bonds but with different donor groups (imino C–H group in the **13tfib** cocrystal and aromatic C–H in the **135tfib** cocrystal). This difference causes a closer approach of the neighboring nitro groups in the crystal structure of (**3**)(**135tfib**) and consequently a noticeably shorter pnictogen bond (*d*(N···O) = 2.88(1) Å).

**Figure 6:**
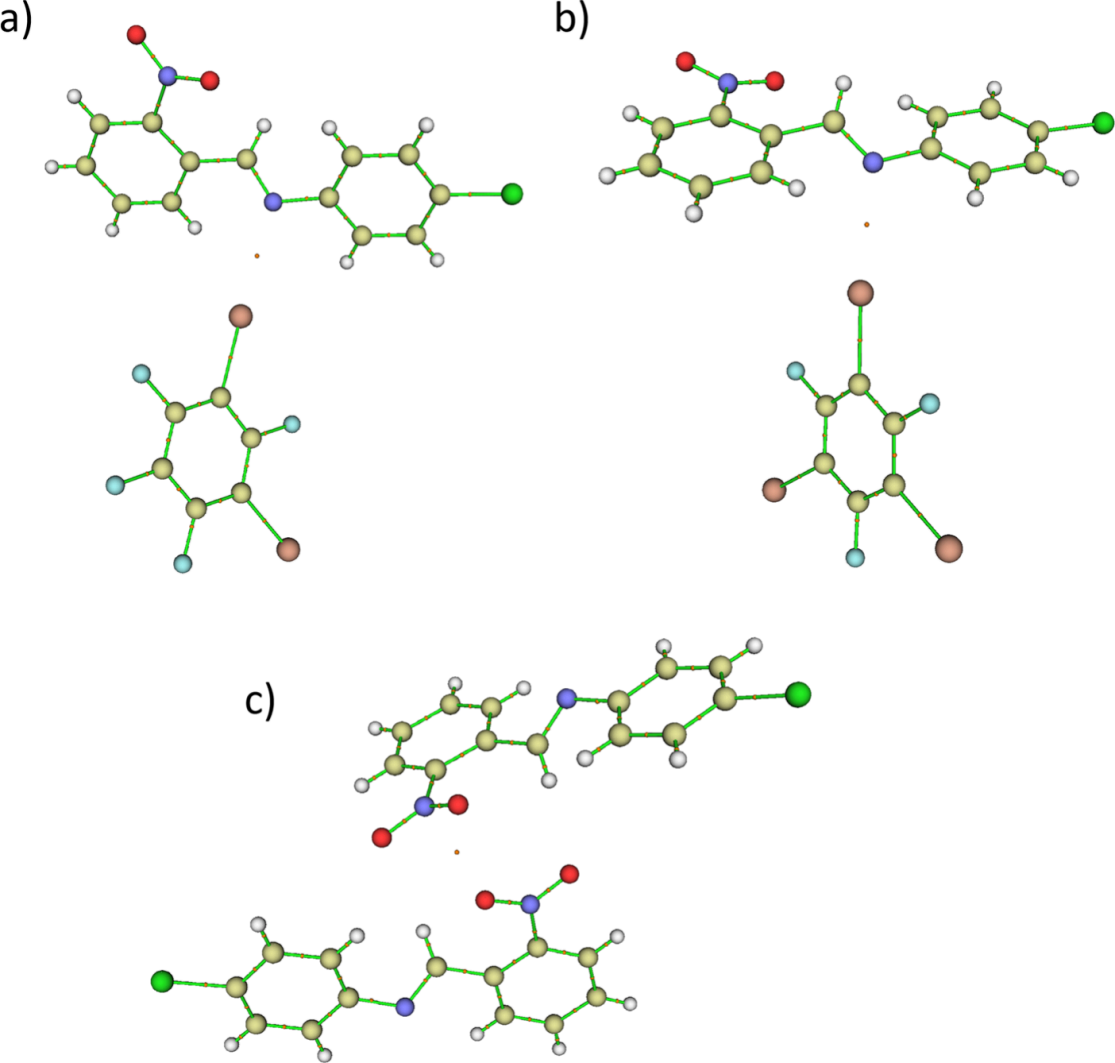
(a) Halogen bond critical points in a donor···acceptor supramolecular complex from cocrystal (**3**)(**13tfib**). (b) Halogen bond critical points in a donor···acceptor supramolecular complex from cocrystal (**3**)(**135tfib**). (c) Pnictogen bond critical points in a donor···acceptor supramolecular complex from cocrystal (**3**)(**135tfib**).

The AIM analysis of the electron density has demonstrated that the NO_2_···NO_2_ pnictogen bonding contacts in all six cases correspond to well-defined bond paths, with electron density at the bond critical point along the N···O path generally between 5.6 × 10^–3^ e Å^–3^ and 5.9 × 10^–3^ e Å^–3^, the only considerable outliers being (**1**)(**135tfib**) with 4.897 × 10^–3^ e Å^–3^ and (**3**)(**135tfib**) with 8.306 × 10^–3^ e Å^–3^. The critical point electron density decreases with increasing bond length. In comparison with the critical point electron densities corresponding to the NO_2_···NO_2_ contacts (Figure 6c, Figures S48–S52), the critical point electron densities of I···N halogen bonding contacts (Figure 6a,b, Figures S44–S47) are generally 2–3 times larger (in the 1.79 × 10^–2^ e Å^–3^ and 1.94 × 10^–2^ e Å^–3^ range). While this seems to indicate that the halogen bonds are dominant interactions in the structures of the cocrystals, the intermolecular interaction energies between the Schiff base molecules are generally 3–8 kJ mol^–1^ (20–40%) greater than those between the imine molecules and halogen donors.

**Figure 7:**
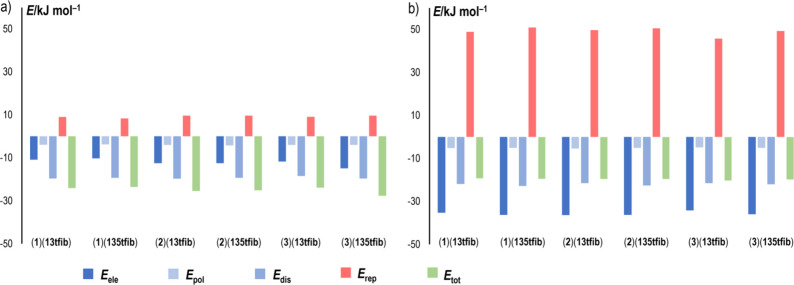
Energy contributions (electrostatic *E*_ele_, polarization *E*_pol_, dispersive *E*_dis_, repulsive *E*_rep_, and total energies *E*_tot_) obtained from interaction energy calculations using Crystal Explorer for interactions between (a) pnictogen-bonded imine molecules and (b) halogen-bonded imine-donor pairs in the six imine-donor cocrystals.

When separate contributions to the overall interaction energies are observed, it can be seen that generally all the attractive contributions (electrostatic *E*_ele_, polarization *E*_pol_, and dispersive *E*_dis_) to the total interaction energy are greater for the halogen-bonded molecules than for pnictogen-bonded imine molecules (Figure 7, Table S3). The difference is most pronounced for the *E*_ele_, which for the interaction between the imine and the halogen bond donor, exceeds the one for the interaction between (pnictogen-bonded) imine molecules by 21–26 kJ mol^–1^, while for *E*_pol_, the difference is in the range 1–1.5 kJ mol^–1^, and for *E*_dis_, it is 1.8–3.5 kJ mol^–1^. This however is compensated by a much larger repulsive contribution (*E*_rep_) for the imine-halogen bond donor pairs, which leads to a lower total interaction energy.

The energetic favorability of the interaction between imine molecules, which includes the NO_2_···NO_2_ pnictogen interactions, leads to an interesting question: why are the NO_2_···NO_2_ pnictogen interactions present only in the six cocrystals (in which also the halogen bonds were established) but are absent in the crystal structures of all three imines? To determine whether this is a peculiarity of this particular system, or it may be indication of a more general trend, we performed a more detailed Cambridge Crystallographic Database (CSD)^57^ search. The CSD survey has revealed that out of 25,787 organic structures that include a nitro group (bound to a carbon atom), 3056 (11.9%) have NO_2_···NO_2_ pnictogen bonding contacts. To investigate the potential effect of competition of this interaction with the halogen bond, a subset of the compounds that along the nitro group also have an iodine atom (bound to a carbon) was investigated. This has yielded a much smaller subset of only 309 structures, in 128 of which (41.4%), the nitro group oxygen acts as a halogen bond acceptor, indicating that there is a potential for competition between the two interactions. However, rather surprisingly, the incidence of NO_2_···NO_2_ pnictogen bonding contacts within this subset was found to be somewhat higher (43 structures, 13.9%) than in the rest of the set of nitro compounds. A possible reason might be that participation of a nitro oxygen in a halogen bond as an acceptor could be expected to increase the positive charge on the π-hole of the corresponding nitrogen atom. However, the incidence of NO_2_···NO_2_ pnictogen bonding contacts among structures where the nitro group is an acceptor of the halogen bond (17 out of 128 structures; 13.1%) is not higher than the incidence among those where there is no C–I···O contact involving the nitro group (26 out of 181 structures; 14.4%).

As the overall number of structures with a potential C–I group as a potential halogen bond donor is rather small, we have extended our database survey onto structures with (considerably more numerous) potential hydrogen bond donors (N–H and O–H groups). There are total of 12,333 entries in the CSD, which comprise both a nitro group and at least one of the said hydrogen donor groups (N/O–H···O=N contacts occurring in 3670, i.e., 29.8%), with the NO_2_···NO_2_ pnictogen bonding contacts occurring in 1520 (12.3%) of them, while in the remaining 13454 nitro-compounds, such contacts can be found in 1545 (11.5%), again showing a higher occurrence of NO_2_···NO_2_ contacts when potentially competing donors are present! Here, however, the argument of the electronic effect of the hydrogen bond on the nitro group does seem to hold as in the case of the 3670 structures where the nitro group is an acceptor of the hydrogen bond. The NO_2_···NO_2_ contacts are present in 643, giving an incidence of 17.5%, while when the hydrogen bond is absent, the incidence of NO_2_···NO_2_ contacts is only 10.1% (877 out of 8663 structures). Contrarily, the presence of a halogen bond donor seems to increase the probability of nitro groups forming NO_2_···NO_2_ contacts regardless of whether the halogen bond donor interacts with the nitro group or not. While the percentages of the occurrence of NO_2_···NO_2_ contacts in structures with C–I halogen bond donors ought to be taken with a pinch of salt due to the rather small sample size (only 309 total structures with both nitro and iodo substituents), it should be noted that they are in agreement with the intermolecular binding in the structures of the six cocrystals prepared within this work, all of which (unlike the pure imines) exhibit NO_2_···NO_2_ contacts, even though the I···O(nitro) halogen bonds are absent.

## Conclusions

In the crystal structures of the three 2-nitrobenzylidene-4′-haloanilines used in this study, the NO_2_···NO_2_ pnictogen bonding is absent, and the halogen atoms of the imines do not participate in halogen bonding either with the nitro oxygen or the imine nitrogen atoms (the two most negative sites on the tested imine molecules) but rather participate only in interhalogen contacts. Introduction of coformers **13tfib** and **135tfib** leads to formation of 1:1 cocrystals where in all six cases, the halogen bond is formed with the imine nitrogen atom rather than with the more negative nitro oxygen. On the other hand, in all six cocrystals, this also leads to close contacts of nitro groups of neighboring imine molecules and formation of NO_2_···NO_2_ pnictogen bonding contacts. The preference of the halogen bond donors for binding to the imine nitrogen rather than the nitro oxygen can probably be attributed to softness of the imine nitrogen as a Lewis base makes it a good acceptor for the iodine (soft acid), allowing for a higher degree of electron exchange between them (as reflected in the critical point electron densities), leaving oxygen and nitrogen atoms of a nitro group to form interactions with nitrogen and oxygen atoms of a neighboring nitro group, respectively. However, the CSD statistics seems to indicate that there might be some general connection between the presence of halogen bonds and the frequency of occurrence of NO_2_···NO_2_ contacts, which seems to merit further investigation.

## Experimental Section

### Synthesis

All starting substances were purchased from commercial sources.

Imines **1**, **2**, and **3** were prepared by condensation reactions of 2-nitrobenzaldehyde (604.0 mg, 4.000 mmol) and 4-haloaniline (halo = Cl, Br, and I; 4.000 mmol) from hot methanol. The crude products were filtered under a vacuum. The resulting products were characterized by powder and single-crystal X-ray diffraction (for **2** and **3**), thermogravimetric analysis, and differential scanning calorimetry. Details on imine synthesis are given in the SI. Powder patterns of imines calculated from single crystal data are in good agreement with measured PXRD patterns of the compounds obtained from the synthesis in solution, which indicates that all prepared compounds are pure phases (see the SI).

### Mechanochemical Cocrystal Screening

Cocrystal screening was performed by grinding mixtures of imines with **13tfib** or **135tfib** in a stoichiometric ratio 1:1. The reaction mixtures (60 mg) were placed in 5 mL stainless steel jars along with 15 μL of acetone and two stainless steel balls 5 mm in diameter and then milled for 30 min in a Retsch MM200 Shaker Mill operating at 25 Hz. The resulting powders were characterized by powder X-ray diffraction, thermogravimetric analysis, and differential scanning calorimetry. Details on mechanochemical experiments are given in the SI.

### One-Pot Mechanochemical Synthesis

The mixtures (60 mg) of 2-nitrobenzaldehyde, 4-haloaniline (halo = Cl, Br, and I), and **13tfib** or **135tfib** were placed in 5 mL stainless steel jars along with 15 μL of acetone and two stainless steel balls 5 mm in diameter and then milled for 30 min in a Retsch MM200 Shaker Mill operating at 25 Hz. The resulting powders were characterized by powder X-ray diffraction. Details on one-pot mechanochemical experiments are given in the SI.

### Single Crystal Preparation

Cocrystals suitable for single-crystal experiments were prepared by crystallization from various solvents. The mixtures (50 mg) of imines and **13tfib** or **135tfib** in 1:1 stoichiometric ratio were dissolved in hot organic solvents. The crystals were obtained by the slow evaporation of the solvents at room temperature. Details on crystallization experiments are given in the SI.

### Powder X-ray diffraction (PXRD)

PXRD experiments were performed on a Malvern PANalytical Aeris Research Edition X-ray diffractometer with CuKα1 (1.54056 Å) radiation at 15 mA and 40 kV. The scattered intensities were measured with a scintillation counter. The angular range was from 5 to 40° (2θ) with steps of 0.0054332° (2θ), and the measuring time was 10.2 s per step. Data analysis was performed using the program package Data Viewer 1.9a.^58^ PXRD patterns are given in the SI (Figures S9–S21).

### Single-Crystal X-ray Diffraction (SCXRD)

The crystal and molecular structures of the prepared imines (**2** and **3**) and cocrystals were determined by single-crystal X-ray diffraction. Diffraction measurements were made on a Rigaku Synergy XtaLAB X-ray diffractometer equipped with a Dualflex source using MoKα radiation, λ = 0.71073 Å. The data sets were collected by using the ω scan mode over the 2θ range up to 64° (Synergy XtaLAB). The data were collected at 170 K. Programs CrysAlis CCD, CrysAlis RED, and CrysAlisPro were employed for data collection, cell refinement, and data reduction.^59^ The structures were solved by direct methods and refined using the SHELXT and SHELXL programs.^60,61^ The structural refinement was performed on *F*^2^ by using all data. Nonhydrogen atoms were refined anisotropically, and hydrogen atoms were placed in calculated positions and treated as riding on their parent atoms. Single-crystal diffraction data for (**3**)(**13tfib**) and (**3**)(**135tfib**) were treated during data reduction as resulting from a combination of two twin components. All calculations were performed using the Olex2 crystallographic suite of programs.^62^ The molecular structures of compounds and their molecular packing projections were prepared by Mercury 2022.3.0.^63^ Details of data collection and crystal structure refinement are listed in Tables S1 and S2 of the SI. Molecular structures showing the atom-labeling schemes are given in the SI (Figures S1–S8). Further details are available from the Cambridge Crystallographic Centre (CCDC 2325894–2325901 contain the crystallographic data for this paper).

### Thermogravimetric Analysis (TGA)

TGA measurements were performed on a Mettler-Toledo TGA/DSC 3+ module. The samples were placed in open 70 μL alumina pans and heated from 25 to 400 °C for **1**, 25 to 320 °C for **2**, **3**, and (**2**)(**13tfib**), and 25 to 300 °C for the rest of the cocrystals at a rate of 10 °C min^–1^ under nitrogen flow of 50 mL min^–1^. The data collection and analysis were performed using the program package STARe Software v16.30.^64^ TGA curves are given in the SI (Figures S22–S31).

### Differential Scanning Calorimetry (DSC)

DSC measurements were performed on a TA Instruments Discovery DSC 25. The samples were placed in hermetically sealed 40 μL TA zero aluminum pans and heated from 25 °C to temperatures a few °C above the melting point at a rate of 10 °C min^–1^ under nitrogen flow of 50 mL min^–1^. Data collection and analysis were performed using the program package TRIOS Software v5.1.1.^65^ DSC spectra are given in the SI (Figures S32–S41).

### Cambridge Structural Database (CSD) Survey

The database search was performed on the CSD version 5.44 (April 2023) with one update.^57^ The searches were restricted to organic substances determined from single crystal data, with exclusion of structures without determined 3D coordinates or with errors as well as ionic or polymeric structures.

### Computational Studies

All calculations were performed using the Gaussian 16 software package.^66^ Geometry optimizations were performed using the M062X/def2-tzvp^67^ level of theory with ultrafine integration grid (99 radial shells and 590 points per shell). The default Gaussian convergence criteria were used. Harmonic frequency calculations were performed on the optimized geometries to ensure the success of each geometry optimization. The figures were prepared using GaussView 6.1.^68^ Interaction energies were calculated by Crystal Explorer (version 21.5, Revision: 608bb32) software,^69^ based on atom positions derived from the determined crystals structures at the B3LYP/6-31G(d,p) level of theory. AIM analysis was performed using the Multiwfn 3.8 software package.^70^ Geometries of pnictogen- and halogen-bonded supramolecular dimers used for AIM analysis were taken from the corresponding crystal structures.

## Supporting Information Available

Experimental details; single-crystal diffraction data for imines **2** and **3** and cocrystals; molecular structures showing the atom-labeling scheme for asymmetric units; PXRD patterns; TGA curves; DSC curves; table with energy contributions for interactions between pnictogen-bonded imine molecules and halogen-bonded imine-donor pairs in the six imine-donor cocrystals; supramolecular networks of imine **2**; interconnection of halogen-bonded chains via I···F interactions into the 3D network in (**1**)(**135tfib**) viewed along the *b* axis; bond critical points in a halogen-bonded donor···acceptor supramolecular complex from cocrystals; bond critical points in a pnictogen-bonded donor···acceptor supramolecular complex from cocrystals (PDF)
