# Supplementary material for: Evaluation of Concomitant Halogen and Pnictogen Bonds in Cocrystals of Imines Derived from 2-Nitrobenzaldehyde and 4-Haloaniline
Source: Cryst Growth Des. 2024 Mar 22;24(7):3010–20. doi: 10.1021/acs.cgd.4c00102 (PMC10996288; doi:10.1021/acs.cgd.4c00102)
Supplement: Supplementary file 1 — cg4c00102_si_001.pdf [file cg4c00102_si_001.pdf]

SUPPORTING INFORMATION

Evaluation of concomitant halogen and pnictogen bonds in cocrystals of imines derived from 2-nitrobenzaldehyde and 4-haloaniline

Nea Baus Topić[a], Nikola Bedeković[a], Leon Poljanić[a,†], Vladimir Stilinović[a], Dominik Cinčić[a*]*

[a] Department of Chemistry, Faculty of Science, University of Zagreb, Horvatovac 102a, 10000 Zagreb, Croatia.

Email: vstilinovic@chem.pmf.hr, dominik@chem.pmf.hr

Table of Contents

Experimental details	Syntheses of imines.	2
	Mechanochemical syntheses of cocrystals.	3
	One-pot mechanochemical syntheses of cocrystals.	4
	Crystallization from solution.	6
Table S1.	Crystal data and refinement details for the prepared imines.	8
Table S2.	Crystal data and refinement details for the prepared cocrystals.	9
Figures S1.–S8.	Molecular structures of imines and cocrystals showing the atom-labeling schemes. Displacement ellipsoids are drawn at the 50 % probability level, and H atoms are shown as small spheres of arbitrary radius.	11
Figures S9.–S21.	PXRD patterns.	15
Figures S22.–S31.	TGA curves.	22
Figures S32.–S41.	DSC curves.	27

Table S3.	Energy contributions (electrostatic E_{ele}, polarisation E_{pol}, dispersive E_{dis}, repulsive E_{rep} and total energies E_{tot}) for interactions between pnictogen-bonded imine molecules and halogen bonded imine-donor pairs in the six imine-donor cocrystals.	32
Figure S42.	Supramolecular networks of imine **2**.	33
Figure S43.	Interconnection of halogen-bonded chains via I···F interactions into 3D network in (**1**)(**135tfib**) viewed along the *b* axis.	33
Figures S44.–S47.	Bond critical points in halogen-bonded donor···acceptor supramolecular complex from cocrystals.	34
Figures S48.–S52.	Bond critical points in halogen-bonded donor···acceptor supramolecular complex from cocrystals.	36

EXPERIMENTAL DETAILS

SYNTHESES OF IMINES

Preparation of **1**

2-nitrobenzaldehyde (604.0 mg, 4.000 mmol) and 4-iodoaniline (876.1 mg, 4.000 mmol) was dissolved in 5.0 mL of hot methanol. The mixture was left at room temperature and the product was filtered under vacuum. The resulting product (3.200 mmol, η = 80%) was characterized by powder X-ray diffraction, thermogravimetric analysis, and differential scanning calorimetry.

Preparation of **2**

2-nitrobenzaldehyde (604.0 mg, 4.000 mmol) and 4-bromoaniline (688.1 mg, 4.000 mmol) were dissolved in 4.0 mL of hot methanol. The mixture was left at room temperature and the product was filtered under vacuum. The resulting product (3.360 mmol, η = 84%) was characterized by

powder and single-crystal X-ray diffraction, thermogravimetric analysis, and differential scanning calorimetry.

Preparation of **3**

2-nitrobenzaldehyde (604.0 mg, 4.000 mmol) and 4-chloroaniline (510.3 mg, 4.000 mmol) were dissolved in 4.0 mL of hot methanol. The mixture was left at room temperature and the product was filtered under vacuum. The resulting product (3.360 mmol, η = 84%) was characterized by powder and single-crystal X-ray diffraction, thermogravimetric analysis, and differential scanning calorimetry.

Syntheses of cocrystals

Mechanochemical syntheses

Mechanochemical synthesis of (**1**)(**13tfib**)

A mixture of **1** (28.0 mg, 79.6 μmol) and **13tfib** (12.0 μL, 79.6 μmol) was placed in a 5 mL stainless steel jar along with 15 μL of acetone and two stainless steel balls 5 mm in diameter. The reaction mixture was then milled for 30 minutes in a Retsch MM200 Shaker Mill operating at 25 Hz.

Mechanochemical synthesis of (**1**)(**135tfib**)

A mixture of **1** (24.5 mg, 69.6 μmol) and **135tfib** (35.5 mg, 69.6 μmol) was placed in a 5 mL stainless steel jar along with 15 μL of acetone and two stainless steel balls 5 mm in diameter. The reaction mixture was then milled for 30 minutes in a Retsch MM200 Shaker Mill operating at 25 Hz.

Mechanochemical synthesis of (**2**)(**13tfib**)

A mixture of **2** (25.9 mg, 84.9 μmol) and **13tfib** (12.8 μL, 84.9 μmol) was placed in a 5 mL stainless steel jar along with 15 μL of acetone and two stainless steel balls 5 mm in diameter. The reaction mixture was then milled for 30 minutes in a Retsch MM200 Shaker Mill operating at 25 Hz.

Mechanochemical synthesis of (**2**)(**135tfib**)

A mixture of **2** (22.5 mg, 73.6 μmol) and **135tfib** (37.5 mg, 73.6 μmol) was placed in a 5 mL stainless steel jar along with 15 μL of acetone and two stainless steel balls 5 mm in diameter. The reaction mixture was then milled for 30 minutes in a Retsch MM200 Shaker Mill operating at 25 Hz.

Mechanochemical synthesis of (**3**)(**13tfib**)

A mixture of **3** (23.6 mg, 90.6 μmol) and **13tfib** (13.6 μL, 90.6 μmol) was placed in a 5 mL stainless steel jar along with 15 μL of acetone and two stainless steel balls 5 mm in diameter. The reaction mixture was then milled for 30 minutes in a Retsch MM200 Shaker Mill operating at 25 Hz.

Mechanochemical synthesis of (**3**)(**135tfib**)

A mixture of **3** (20.3 mg, 77.9 μmol) and **135tfib** (39.7 mg, 77.9 μmol) was placed in a 5 mL stainless steel jar along with 15 μL of acetone and two stainless steel balls 5 mm in diameter. The reaction mixture was then milled for 30 minutes in a Retsch MM200 Shaker Mill operating at 25 Hz.

ONE-POT MECHANOCHEMICAL SYNTHESES

One-pot mechanochemical synthesis of (**1**)(**13tfib**)

A mixture of 2-nitrobenzaldehyde (11.7 mg, 77.7 μmol), 4-iodoaniline (17.0 mg, 77.7 μmol) and **13tfib** (11.7 μL, 77.7 μmol) was placed in a 5 mL stainless steel jar along with 15 μL of acetone and two stainless steel balls 5 mm in diameter. The reaction mixture was then milled for 30 minutes in a Retsch MM200 Shaker Mill operating at 25 Hz.

One-pot mechanochemical synthesis of (**1**)(**135tfib**)

A mixture of 2-nitrobenzaldehyde (10.3 mg, 68.2 μmol), 4-iodoaniline (14.9 mg, 68.2 μmol) and **135tfib** (34.8 mg, 68.2 μmol) was placed in a 5 mL stainless steel jar along with 15 μL of acetone and two stainless steel balls 5 mm in diameter. The reaction mixture was then milled for 30 minutes in a Retsch MM200 Shaker Mill operating at 25 Hz.

One-pot mechanochemical synthesis of (**2**)(**13tfib**)

A mixture of 2-nitrobenzaldehyde (12.5 mg, 82.8 μmol), 4-bromoaniline (14.2 mg, 82.8 μmol) and **13tfib** (12.5 μL, 82.8 μmol) was placed in a 5 mL stainless steel jar along with 15 μL of acetone and two stainless steel balls 5 mm in diameter. The reaction mixture was then milled for 30 minutes in a Retsch MM200 Shaker Mill operating at 25 Hz.

One-pot mechanochemical synthesis of (**2**)(**135tfib**)

A mixture of 2-nitrobenzaldehyde (10.9 mg, 72.0 μmol), 4-bromoaniline (12.4 mg, 72.0 μmol) and **135tfib** (36.7 mg, 72.0 μmol) was placed in a 5 mL stainless steel jar along with 15 μL of acetone and two stainless steel balls 5 mm in diameter. The reaction mixture was then milled for 30 minutes in a Retsch MM200 Shaker Mill operating at 25 Hz.

One-pot mechanochemical synthesis of (**3**)(**13tfib**)

A mixture of 2-nitrobenzaldehyde (13.3 mg, 88.2 μmol), 4-chloroaniline (11.2 mg, 88.2 μmol) and **13tfib** (13.3 μL, 88.2 μmol) was placed in a 5 mL stainless steel jar along with 15 μL of acetone and two stainless steel balls 5 mm in diameter. The reaction mixture was then milled for 30 minutes in a Retsch MM200 Shaker Mill operating at 25 Hz.

One-pot mechanochemical synthesis of (**3**)(**135tfib**)

A mixture of 2-nitrobenzaldehyde (11.5 mg, 76.1 μmol), 4-chloroaniline (9.7 mg, 76.1 μmol) and **135tfib** (38.8 mg, 76.1 μmol) was placed in a 5 mL stainless steel jar along with 15 μL of acetone and two stainless steel balls 5 mm in diameter. The reaction mixture was then milled for 30 minutes in a Retsch MM200 Shaker Mill operating at 25 Hz.

CRYSTALLIZATION FROM SOLUTION

Crystallization of (**1**)(**13tfib**)

A mixture of **1** (23.4 mg, 66.3 μmol) and 1,3-diiodotetrafluorobenzene (**13tfib**) (10.0 μL, 66.3 μmol) was dissolved in 0.5 mL of hot acetone. The mixture was left to crystallize at room temperature until yellow needle-like crystalline products were obtained.

Crystallization of (**1**)(**135tfib**)

A mixture of **1** (20.4 mg, 58.0 μmol) and 1,3,5-triiodotrifluorobenzene (**135tfib**) (29.6 mg, 58.0 μmol) was dissolved in 0.5 mL of hot dichloromethane. The mixture was left to crystallize at room temperature until yellow prism-like crystalline products were obtained.

Crystallization of (**2**)(**13tfib**)

A mixture of **2** (21.6 mg, 70.7 μmol) and 1,3-diiodotetrafluorobenzene (**13tfib**) (10.5 μL, 70.7 μmol) was dissolved in 0.5 mL of hot acetonitrile. The mixture was left to crystallize at room temperature until yellow prism-like crystalline products were obtained.

Crystallization of (**2**)(**135tfib**)

A mixture of **2** (18.7 mg, 61.4 μmol) and 1,3,5-triiodotrifluorobenzene (**135tfib**) (31.3 mg, 61.4 μmol) was dissolved in 1.0 mL of hot methanol. The mixture was left to crystallize at room temperature until colourless plate-like crystalline products were obtained.

Crystallization of (**3**)(**13tfib**)

A mixture of **3** (19.7 mg, 75.5 μmol) and 1,3-diiodotetrafluorobenzene (**13tfib)** (11.0 μL, 75.5 μmol) was dissolved in 1.0 mL of hot methanol. The mixture was left to crystallize at room temperature until yellow prism-like crystalline products were obtained.

Crystallization of (**3**)(**135tfib**)

A mixture of **3** (16.9 mg, 64.9 μmol) and 1,3,5-triiodotrifluorobenzene (**135tfib)** (33.1 mg, 64.9 μmol) was dissolved in 1.0 mL of hot methanol. The mixture was left to crystallize at room temperature until yellow prism-like crystalline products were obtained.

Table S1. Crystal data and refinement details for the prepared imines.

	2	3
Molecular formula	$C_{13}H_9BrN_2O_2$	$C_{13}H_9ClN_2O_2$
M_r	305.12	260.67
Crystal system	orthorhombic	orthorhombic
Space group	$P2_12_12_1$	$P2_12_12_1$
Crystal data:		
a / Å	3.8644(2)	3.8208(3)
b / Å	6.9893(3)	6.9913(7)
c / Å	43.739(2)	43.123(4)
α / °	90	90
β / °	90	90
γ / °	90	90
V / Å^3	1181.37(10)	1154.32(18)
Z	4	4
D_{calc} / g cm^3	1.715	1.500
λ(MoK_α) / Å	0.71073	0.71073
T / K	170	170
Crystal size / mm^3	0.207 x 0.074 x 0.052	0.25 x 0.08 x 0.037
μ / mm^{-1}	4.718	0.325
F(000)	608.0	536.0
Refl. collected/unique	9602 / 2988	6083 / 2134
Parameters/restraints	163 / 0	163 / 0

$\Delta\rho_{max}$, $\Delta\rho_{min}$ / e Å^{-3}	0.690; –0.584	0.249; –0.294
$R[F^2 > 4\sigma(F^2)]$	0.0360	0.0479
w$R(F^2)$	0.0814	0.1044
Goodness-of-fit, S	1.063	1.015

Table S2. Crystal data and refinement details for the prepared cocrystals.

	(**1**)(**13tfib**)	(**1**)(**135tfib**)
Molecular formula	$C_{19}H_9F_4I_3N_2O_2$	$C_{19}H_9F_3I_4N_2O_2$
M_r	753.98	861.88
Crystal system	orthorhombic	monoclinic
Space group	$P2_12_12_1$	$P2_1$
Crystal data:		
a / Å	4.4022(2)	13.6954(6)
b / Å	13.6156(10)	4.4344(1)
c / Å	35.403(2)	19.1112(6)
α / °	90	90
β / °	90	102.395(4)
γ / °	90	90
V / Å^3	2122.0(2)	1133.59(7)
Z	4	2
D_{calc} / g cm^3	2.360	2.525
λ(MoK_α) / Å	0.71073	0.71073
T / K	170	170
Crystal size / mm^3	0.56 x 0.09 x 0.02	0.402 x 0.135 x 0.062
μ / mm^{-1}	4.472	5.540
F(000)	1392.0	784.0
Refl. collected/unique	11233 / 3721	19063 / 6588
Parameters/restraints	259 / 0	271 / 1
$\Delta\rho_{max}$, $\Delta\rho_{min}$ / e Å^{-3}	2.672; -1.544	0.656; –0.729

$R[F^2 > 4\sigma(F^2)]$	0.0767	0.0368
$wR(F^2)$	0.1872	0.0755
Goodness-of-fit, S	1.015	0.993

Table S2. continued:

	(**2**)(**13tfib**)	(**2**)(**135tfib**)
Molecular formula	$C_{19}H_9BrF_4I_2N_2O_2$	$C_{19}H_9BrF_3I_3N_2O_2$
M_r	706.98	841.88
Crystal system	orthorhombic	monoclinic
Space group	$P2_12_12_1$	$P2_1$
Crystal data:		
a / Å	4.4721(2)	13.3373(6)
b / Å	13.2730(5)	4.4993(2)
c / Å	35.2998(15)	18.9018(8)
α / °	90	90
β / °	90	100.625(4)
γ / °	90	90
V / Å^3	2095.33(15)	1114.82(9)
Z	4	2
D_{calc} / g cm^3	2.241	2.428
λ(MoK_α) / Å	0.71073	0.71073
T / K	170	170
Crystal size / mm^3	0.489 x 0.141 x 0.048	0.402 x 0.135 x 0.062
μ / mm^{-1}	4.962	6.041
F(000)	1320.0	748.0
Refl. collected/unique	15978 / 5032	19363 / 6516
Parameters/restraints	272 / 0	271 / 1
$\Delta\rho_{max}$, $\Delta\rho_{min}$ / e Å^{-3}	0.743; -0.725	0.605; –0.837

$R[F^2 > 4\sigma(F^2)]$	0.0400	0.0319
$wR(F^2)$	0.0879	0.0640
Goodness-of-fit, S	1.043	1.006

Table S2. continued:

	(**3**)(**13tfib**)	(**3**)(**135tfib**)
Molecular formula	$C_{19}H_9ClF_4I_2N_2O_2$	$C_{19}H_9ClF_3I_3N_2O_2$
M_r	662.53	770.43
Crystal system	monoclinic	monoclinic
Space group	$P2_1/c$	$P2_1/n$
Crystal data:		
a / Å	12.8877(8)	21.1629(8)
b / Å	4.2747(2)	4.8189(2)
c / Å	36.9674(14)	22.7470(11)
α / °	90	90
β / °	94.894(4)	109.406(5)
γ / °	90	90
V / Å^3	2029.15(18)	2187.99(17)
Z	4	4
D_{calc} / g cm^3	2.169	2.339
λ(MoK_α) / Å	0.71073	0.71073
T / K	170	170
Crystal size / mm^3	0.631 x 0.301 x 0.101	0.633 x 0.213 x 0.099
μ / mm^{-1}	3.827	4.452
F(000)	1248.0	1424.0
Refl. collected/unique	18706 / 5855	9845 / 8487
Parameters/restraints	271 / 0	272 / 0
$\Delta\rho_{max}$, $\Delta\rho_{min}$ / e Å^{-3}	0.965; -0.804	1.688; –1.486

$R[F^2 > 4\sigma(F^2)]$	0.0455	0.0621
$wR(F^2)$	0.1052	0.1938
Goodness-of-fit, S	1.037	1.048

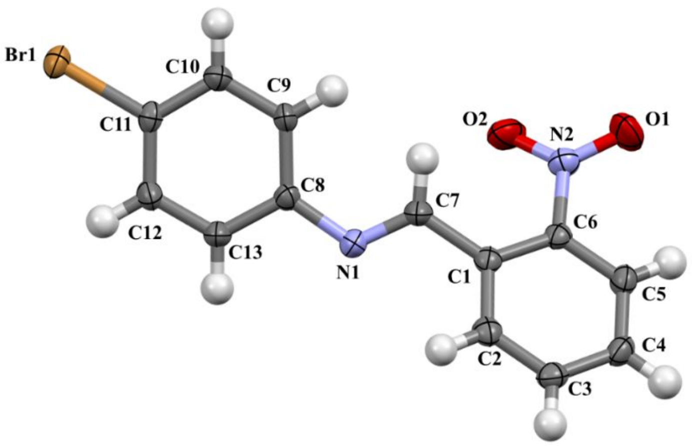

Figure S1. Molecular structure of **2** showing the atom-labelling scheme. Displacement ellipsoids are drawn at the 50 % probability level, and H atoms are shown as small spheres of arbitrary radius.

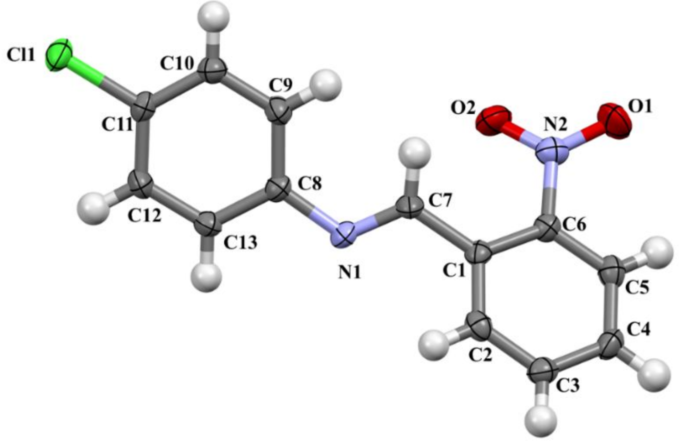

Figure S2. Molecular structure of **3** showing the atom-labelling scheme. Displacement ellipsoids are drawn at the 50 % probability level, and H atoms are shown as small spheres of arbitrary radius.

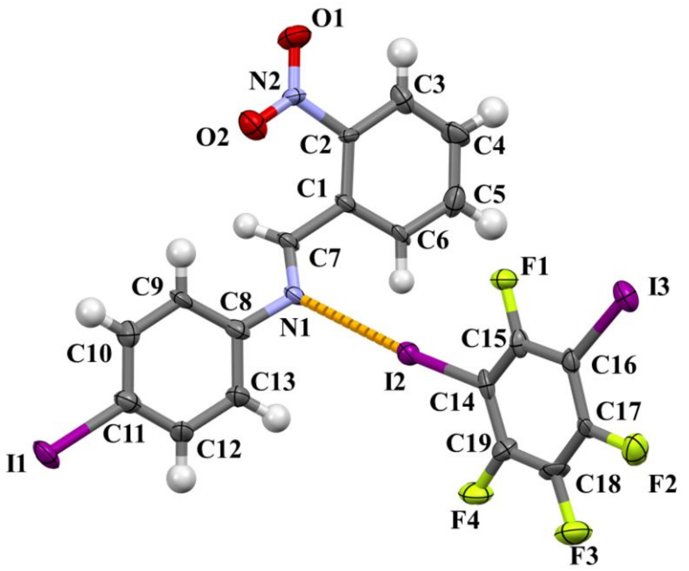

Figure S3. Molecular structure of (1)(**13tfib**) showing the atom-labelling scheme. Displacement ellipsoids are drawn at the 50 % probability level, and H atoms are shown as small spheres of arbitrary radius.

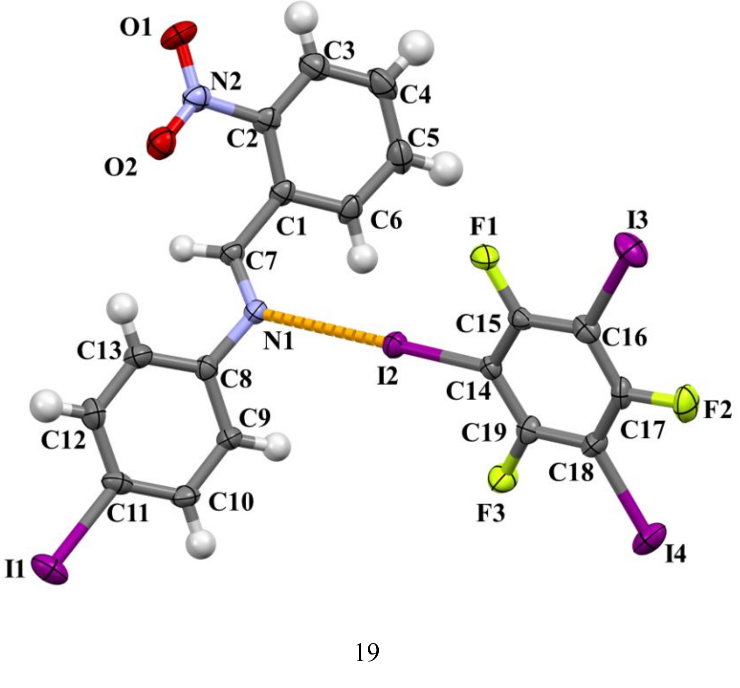

Figure S4. Molecular structure of (1)(**135tfib**) showing the atom-labelling scheme. Displacement ellipsoids are drawn at the 50 % probability level, and H atoms are shown as small spheres of arbitrary radius.

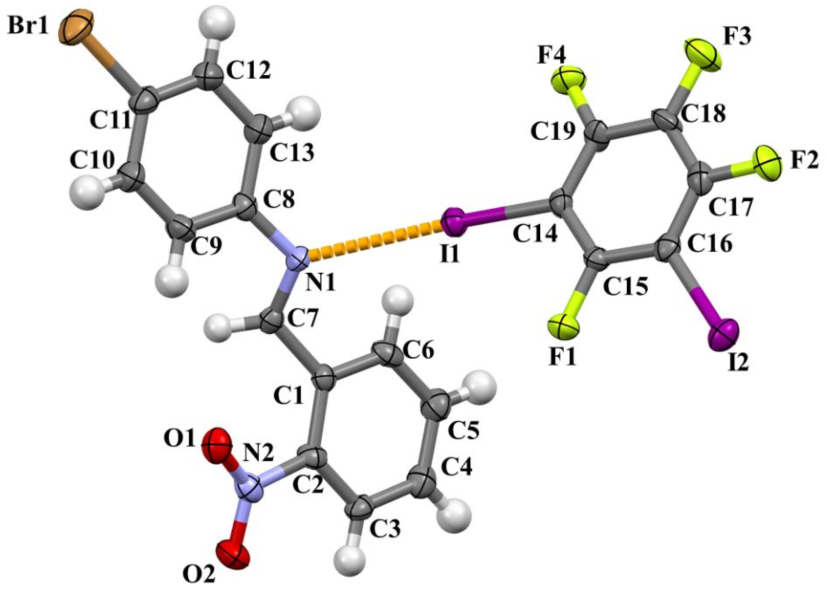

Figure S5. Molecular structure of (**2**)(**13tfib**) showing the atom-labelling scheme. Displacement ellipsoids are drawn at the 50 % probability level, and H atoms are shown as small spheres of arbitrary radius.

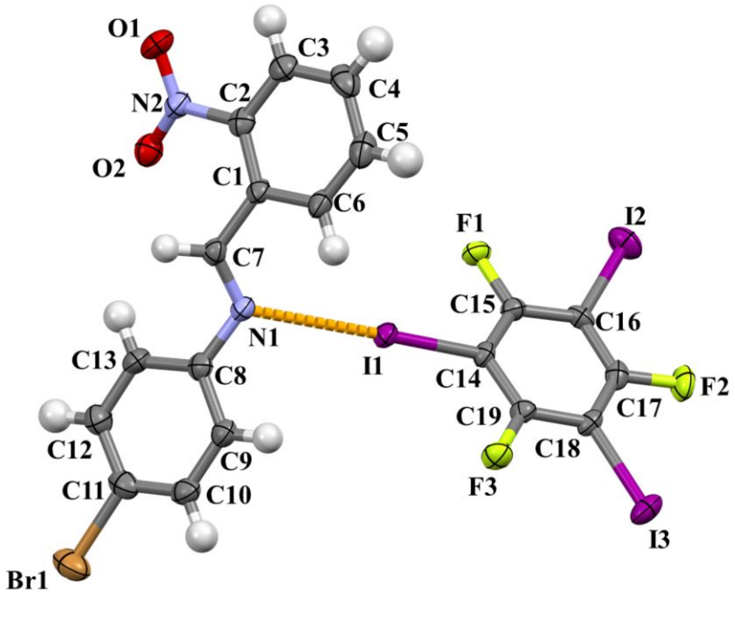

Figure S6. Molecular structure of (**2**)(**135tfib**) showing the atom-labelling scheme. Displacement ellipsoids are drawn at the 50 % probability level, and H atoms are shown as small spheres of arbitrary radius.

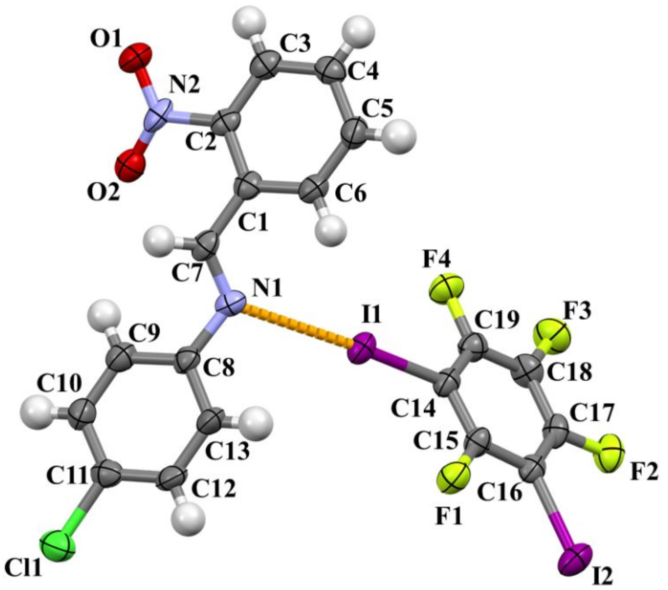

Figure S7. Molecular structure of (**3**)(**13tfib**) showing the atom-labelling scheme. Displacement ellipsoids are drawn at the 50 % probability level, and H atoms are shown as small spheres of arbitrary radius.

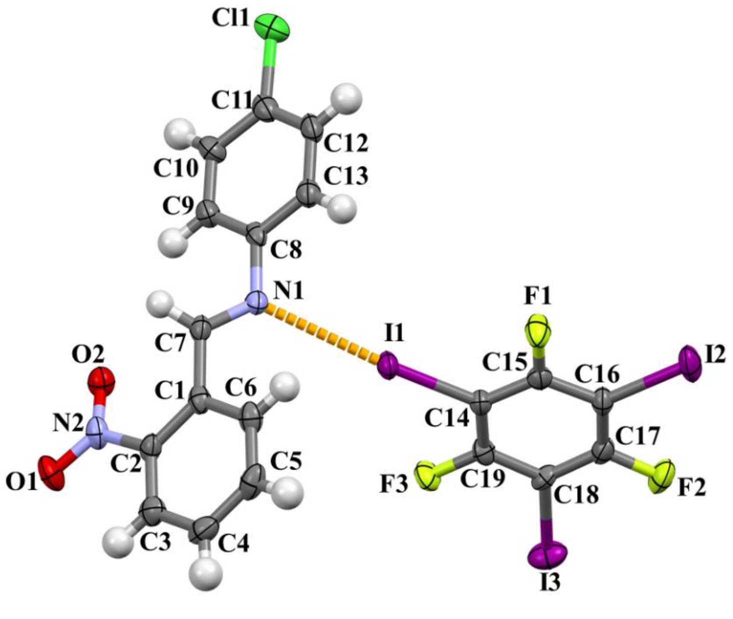

Figure S8. Molecular structure of (**3**)(**135tfib**) showing the atom-labelling scheme. Displacement ellipsoids are drawn at the 50 % probability level, and H atoms are shown as small spheres of arbitrary radius.

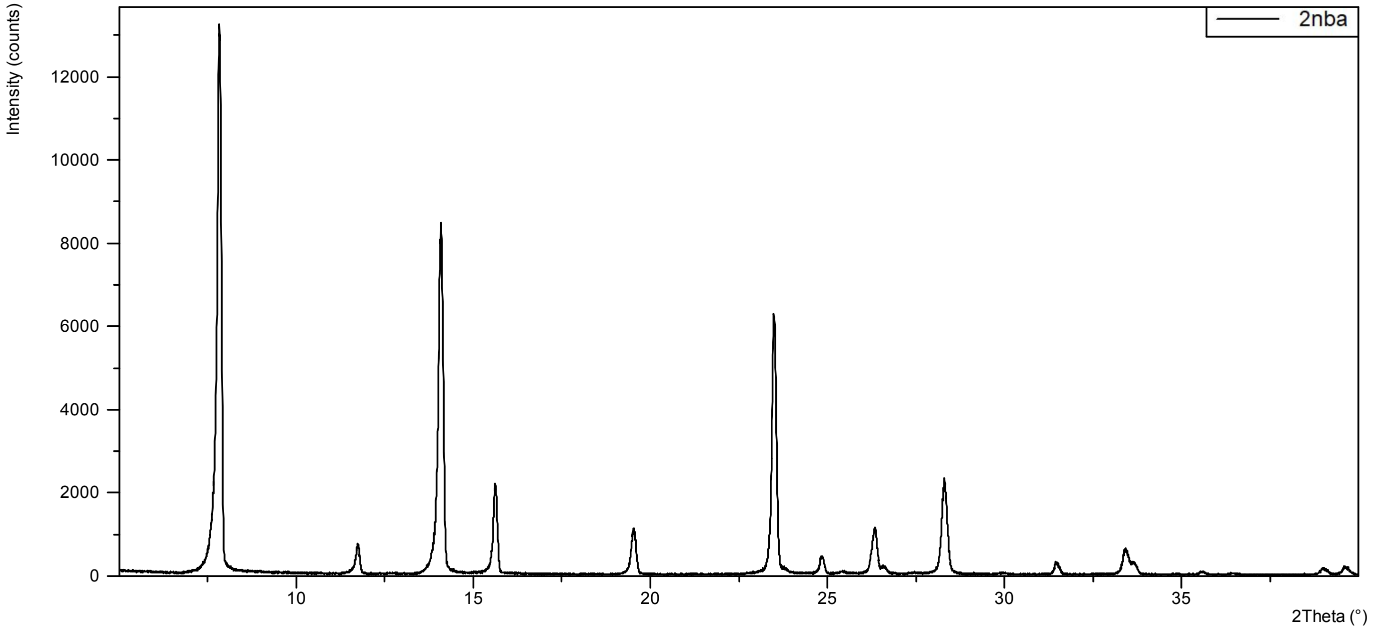

Figure S9. PXRD pattern of 2-nitrobenzaldehyde.

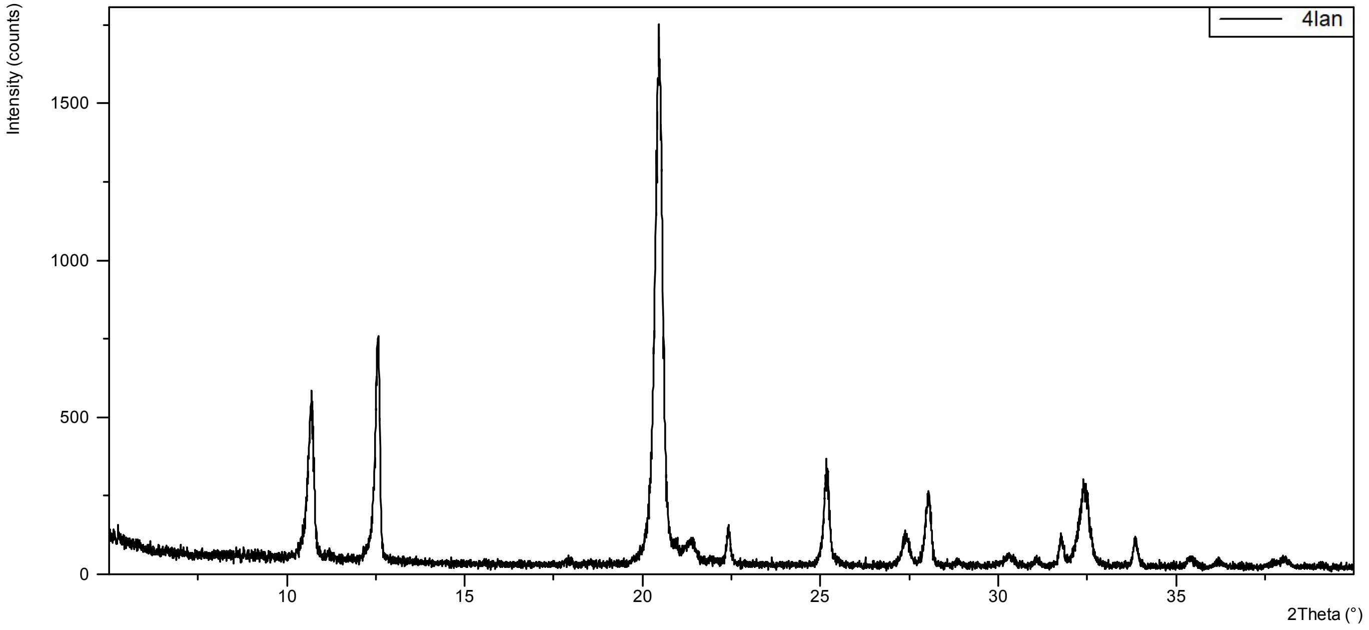

Figure S10. PXRD pattern of 4-iodoaniline.

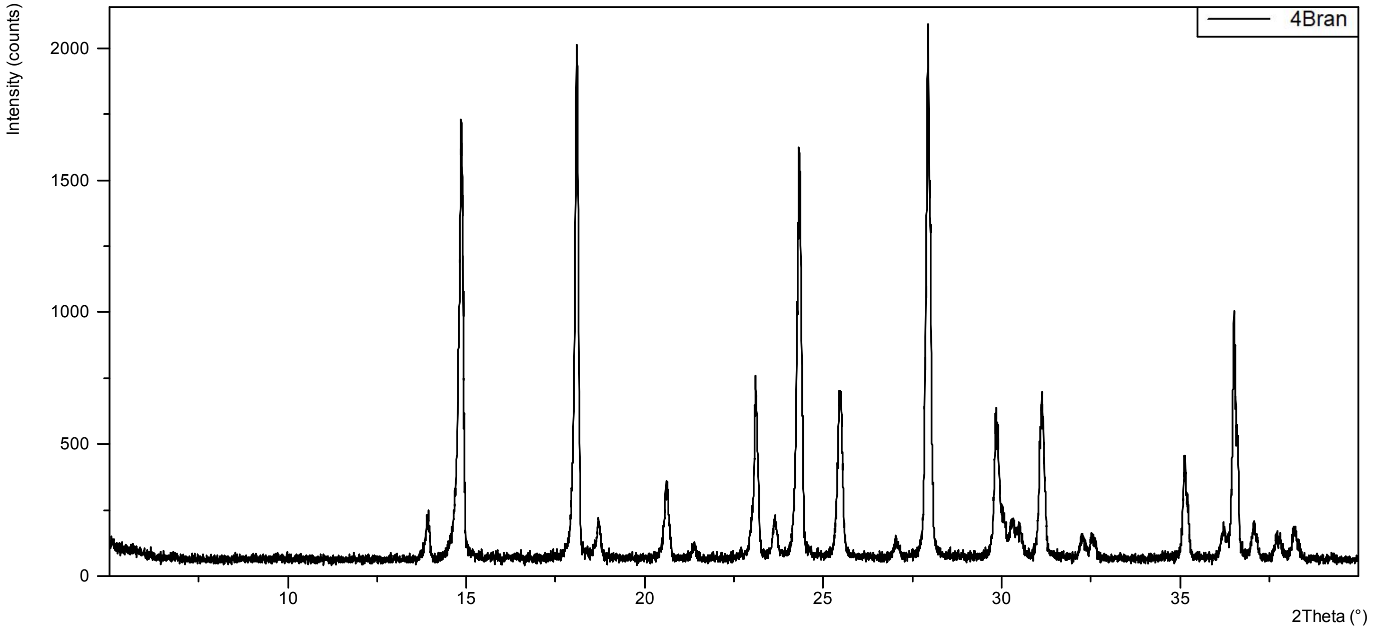

Figure S11. PXRD pattern of 4-bromoaniline.

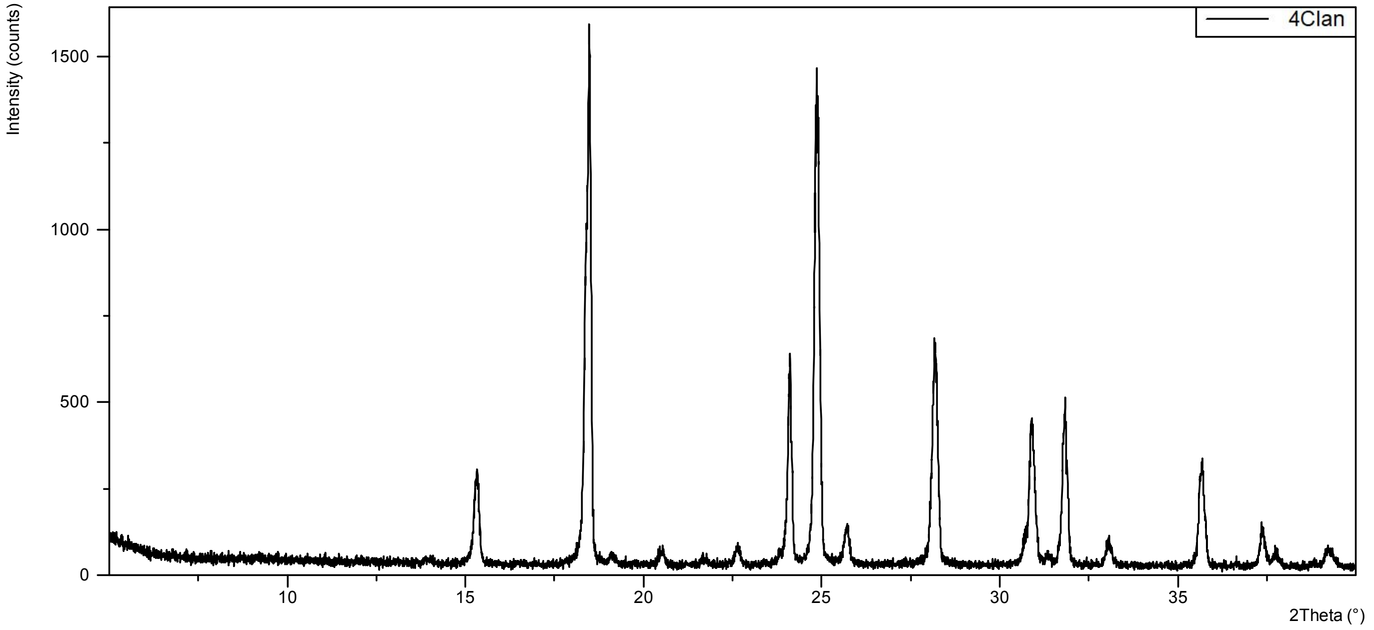

Figure S12. PXRD pattern of 4-chloroaniline.

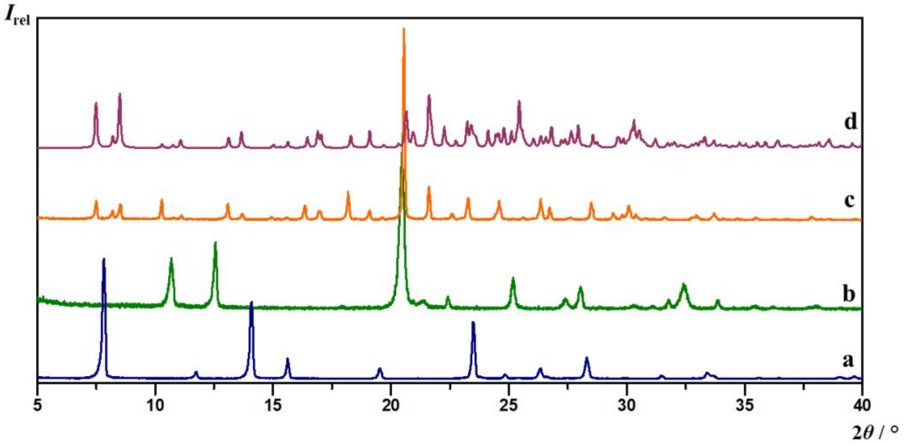

Figure S13. PXRD pattern of a) 2-nitrobenzaldehyde, b) 4-iodoaniline, c) product obtained by solution synthesis from 2-nitrobenzaldehyde and 4-iodoaniline in methanol and d) calculated pattern from **1** single crystal data (refcode: XUDYAW).

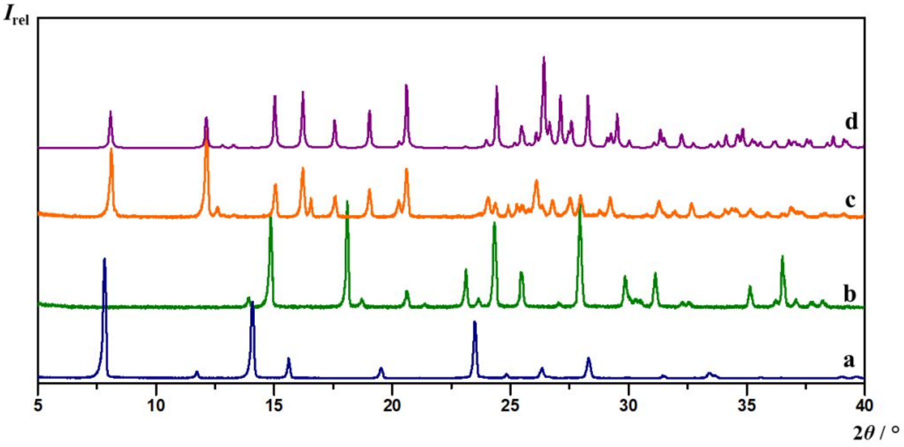

Figure S14. PXRD pattern of a) 2-nitrobenzaldehyde, b) 4-bromoaniline, c) product obtained by solution synthesis from 2-nitrobenzaldehyde and 4-bromoaniline in methanol and d) calculated pattern from **2** single crystal data.

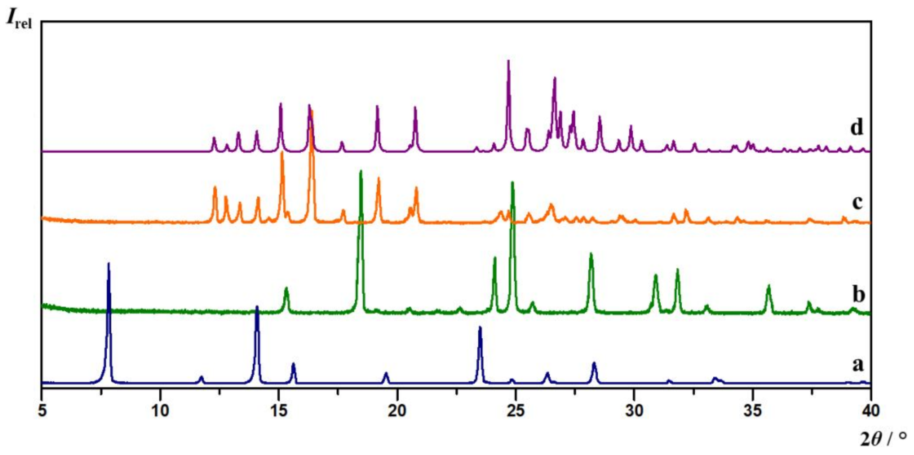

Figure S15. PXRD pattern of a) 2-nitrobenzaldehyde, b) 4-chloroaniline, c) product obtained by solution synthesis from 2-nitrobenzaldehyde and 4-chloroaniline in methanol, d) calculated pattern from **3** single crystal data,

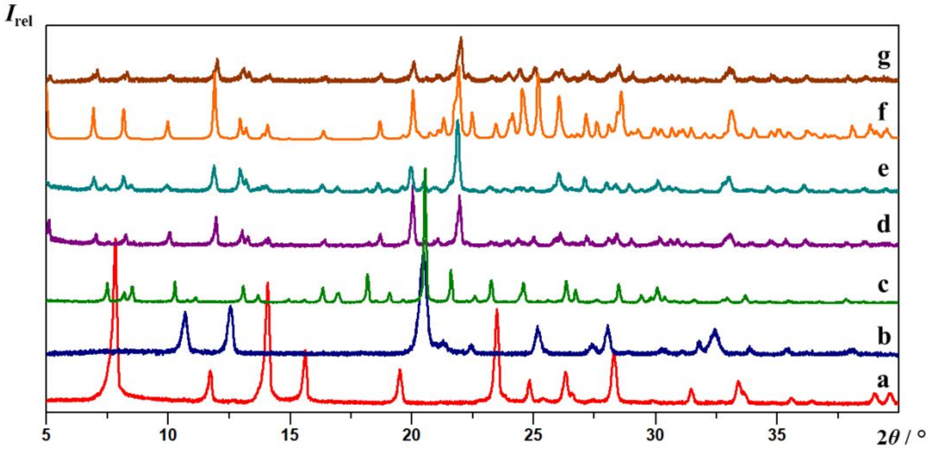

Figure S16. PXRD pattern of a) 2-nitrobenzaldehyde, b) 4-iodoaniline, c) **1**, d) product obtained by acetone-assisted grinding of **1** and **13tfib** in stoichoimetric ratio 1:1, e) product obtained by

solution synthesis from **1** and **13tfib** in acetone, f) calculated pattern from (**1**)(**13tfib**) single crystal data, g) product obtained by acetone-assisted grinding of 2-nitrobenzaldehyde, 4-iodoaniline and **13tfib** in stoichoimetric ratio 1:1:1.

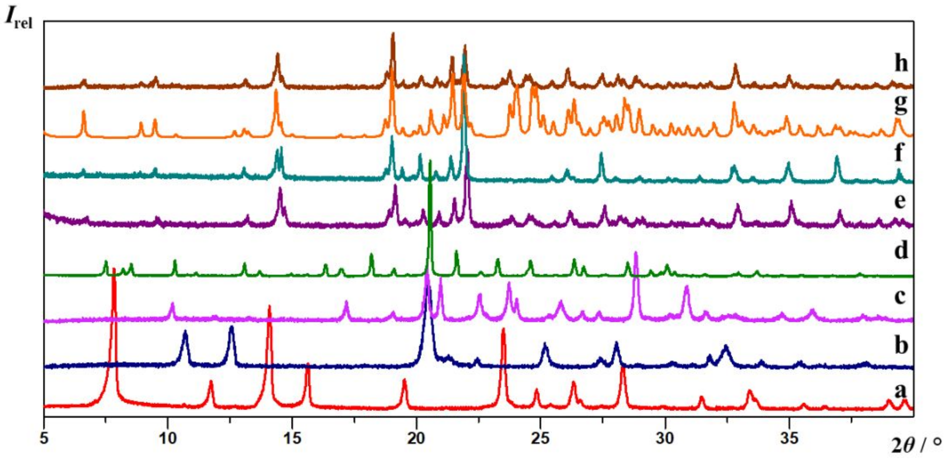

Figure S17. PXRD pattern a) 2-nitrobenzaldehyde, b) 4-iodoaniline, c) **135tfib**, d) **1**, e) product obtained by acetone-assisted grinding of **1** and **135tfib** in stoichoimetric ratio 1:1, f) product obtained by solution synthesis from **1** and **135tfib** in CH_2Cl_2, g) calculated pattern from (**1**)(**135tfib**) single crystal data, h) product obtained by acetone-assisted grinding of 2-nitrobenzaldehyde, 4-iodoaniline and **135tfib** in stoichoimetric ratio 1:1:1.

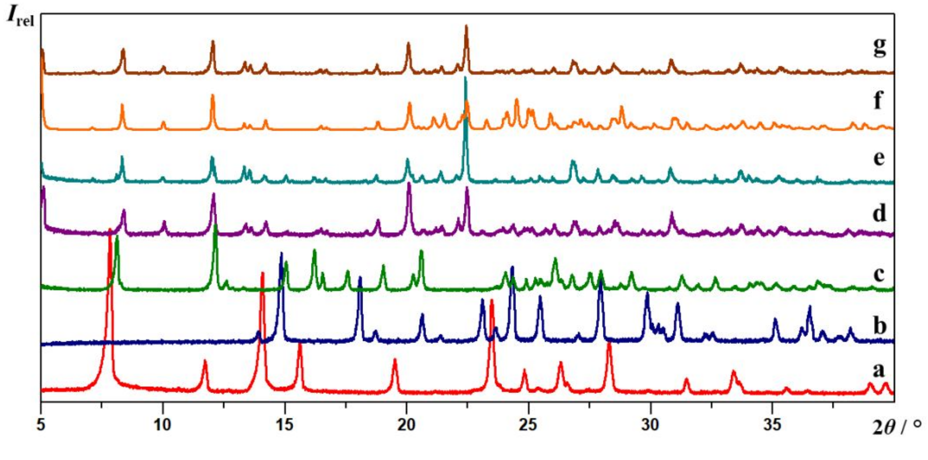

Figure S18. PXRD pattern of a) 2-nitrobenzaldehyde, b) 4-bromoaniline, c) **2**, d) product obtained by acetone-assisted grinding of **2** and **13tfib** in stoichoimetric ratio 1:1, e) product obtained by solution synthesis from **2** and **13tfib** in acetone, f) calculated pattern from (**2**)(**13tfib**) single crystal data, g) product obtained by acetone-assisted grinding of 2-nitrobenzaldehyde, 4-bromoaniline and **13tfib** in stoichoimetric ratio 1:1:1.

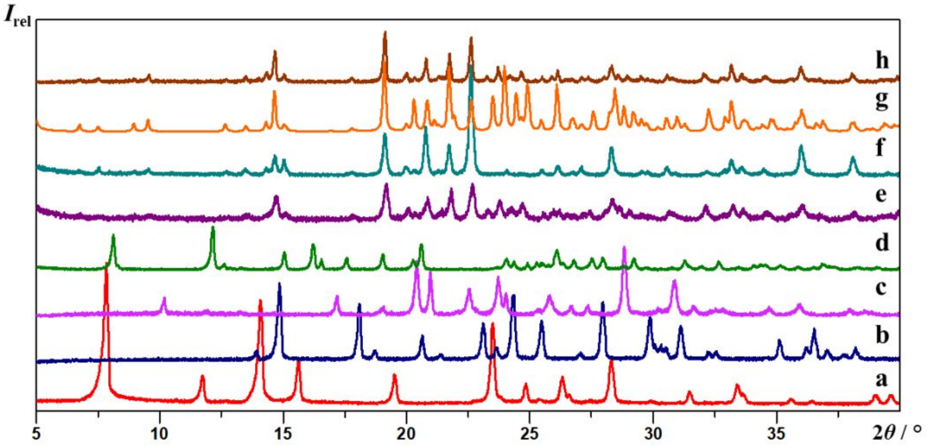

Figure S19. PXRD pattern a) 2-nitrobenzaldehyde, b) 4-bromoaniline, c) **135tfib**, d) **2**, e) product obtained by acetone-assisted grinding of **2** and **135tfib** in stoichoimetric ratio 1:1, f) product obtained by solution synthesis from **2** and **135tfib** in methanol, g) calculated pattern from (**2**)(**135tfib**) single crystal data, h) product obtained by acetone-assisted grinding of 2-nitrobenzaldehyde, 4-bromoaniline and **135tfib** in stoichoimetric ratio 1:1:1.

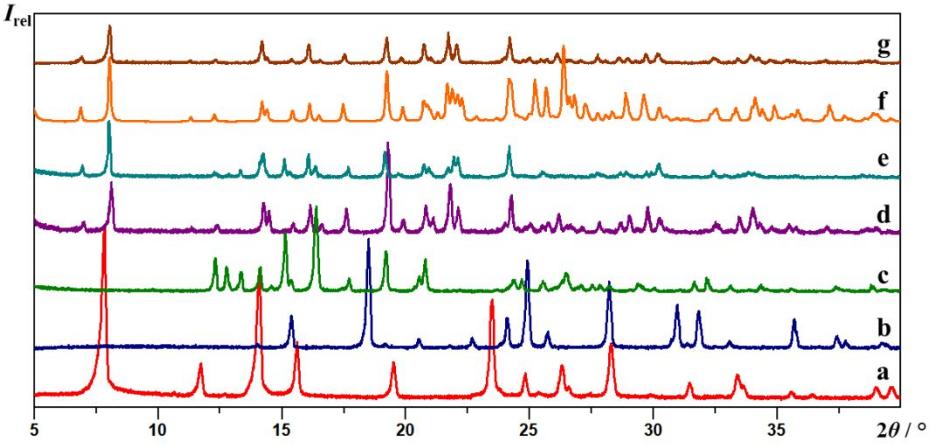

Figure S20. PXRD pattern of a) 2-nitrobenzaldehyde, b) 4-chloroaniline, c) **3**, d) product obtained by acetone-assisted grinding of **3** and **13tfib** in stoichoimetric ratio 1:1, e) product obtained by solution synthesis from **3** and **13tfib** in acetone, f) calculated pattern from (**3**)(**13tfib**) single crystal data, g) product obtained by acetone-assisted grinding of 2-nitrobenzaldehyde, 4-chloroaniline and **13tfib** in stoichoimetric ratio 1:1:1.

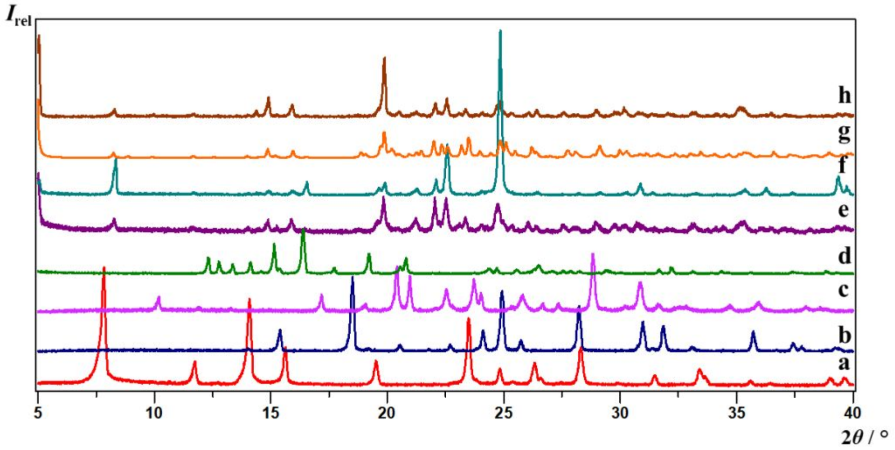

Figure S21. PXRD pattern a) 2-nitrobenzaldehyde, b) 4-chloroaniline, c) **135tfib**, d) **3**, e) product obtained by acetone-assisted grinding of **3** and **135tfib** in stoichoimetric ratio 1:1, f) product obtained by solution synthesis from **3** and **135tfib** in methanol, g) calculated pattern from (**3**)(**135tfib**) single crystal data, h) product obtained by acetone-assisted grinding of 2-nitrobenzaldehyde, 4-chloroaniline and **135tfib** in stoichoimetric ratio 1:1:1.

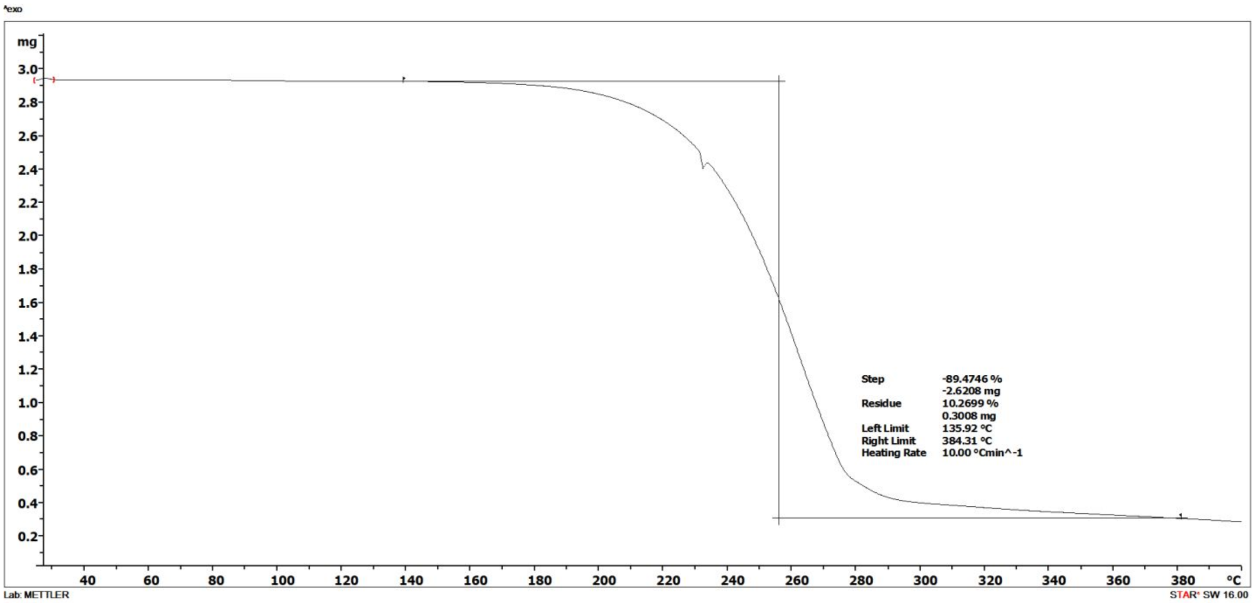

Figure S22. TGA curve of **1**.

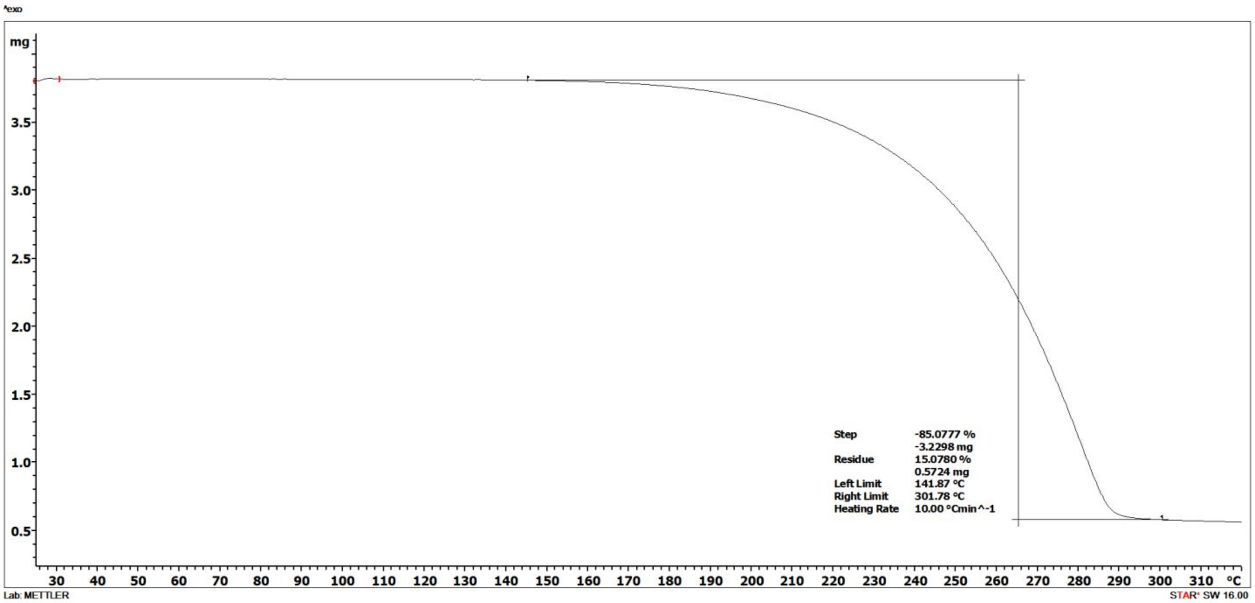

Figure S23. TGA curve of **2**.

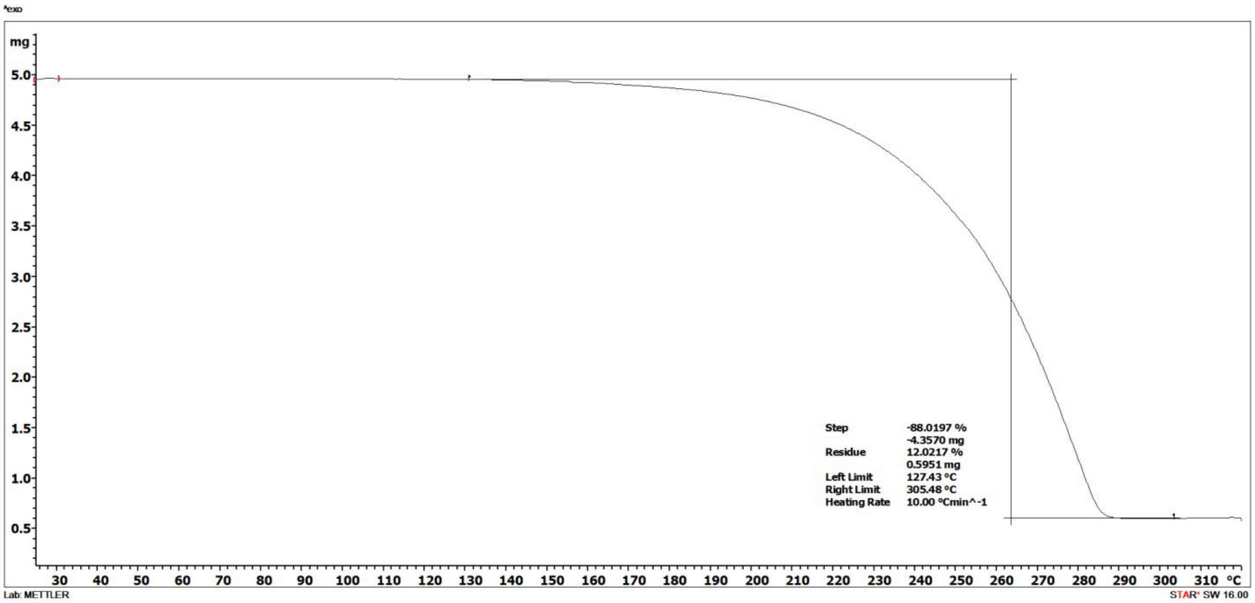

Figure S24. TGA curve of **3**.

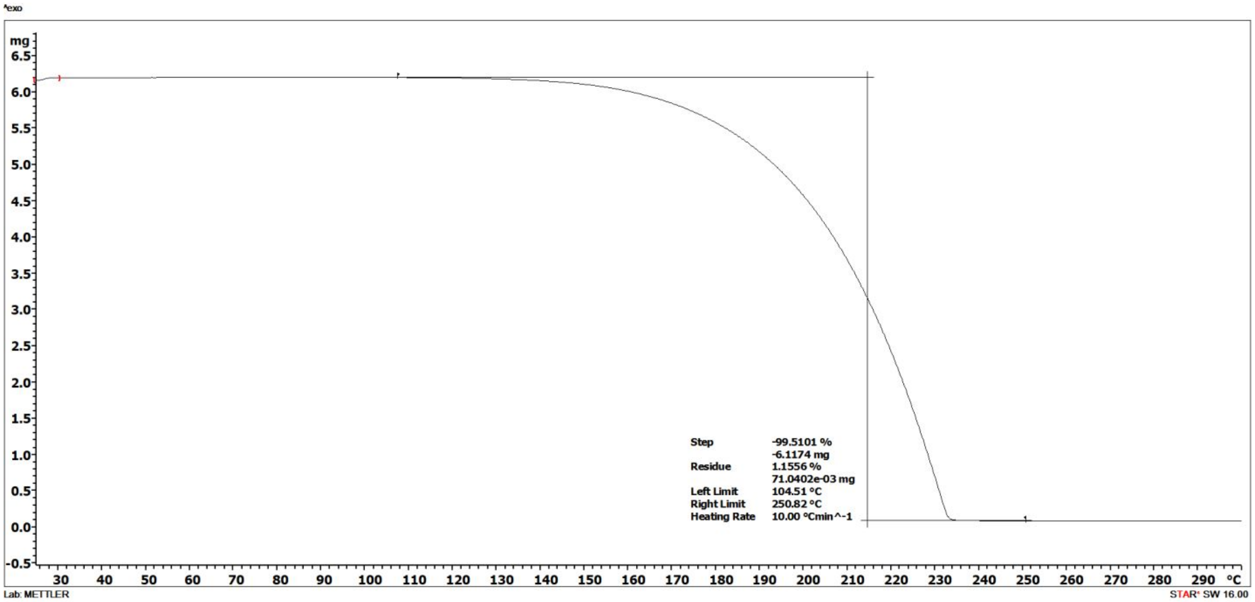

Figure S25. TGA curve of **135tfib**.

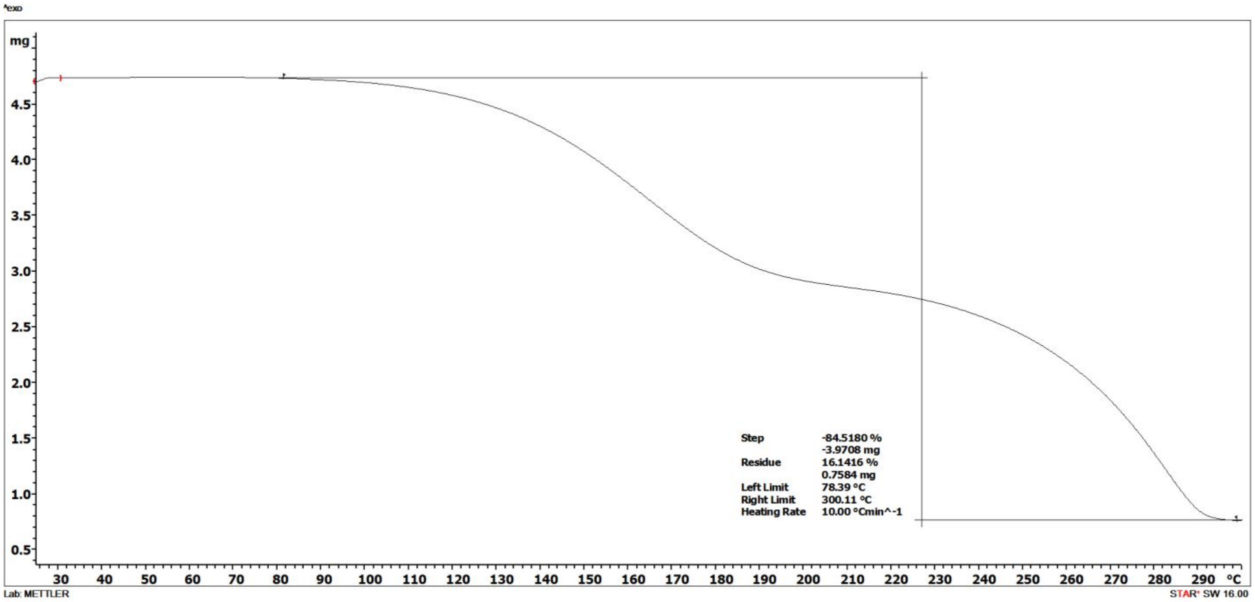

Figure S26. TGA curve of (**1**)(**13tfib**).

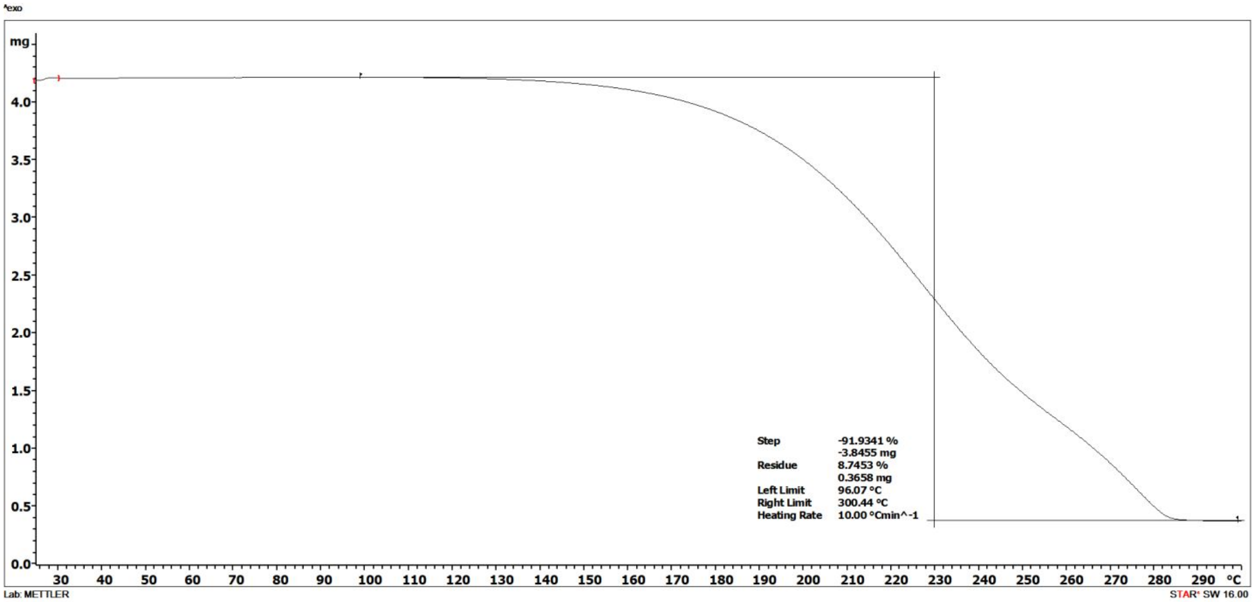

Figure S27. TGA curve of (**1**)(**135tfib**).

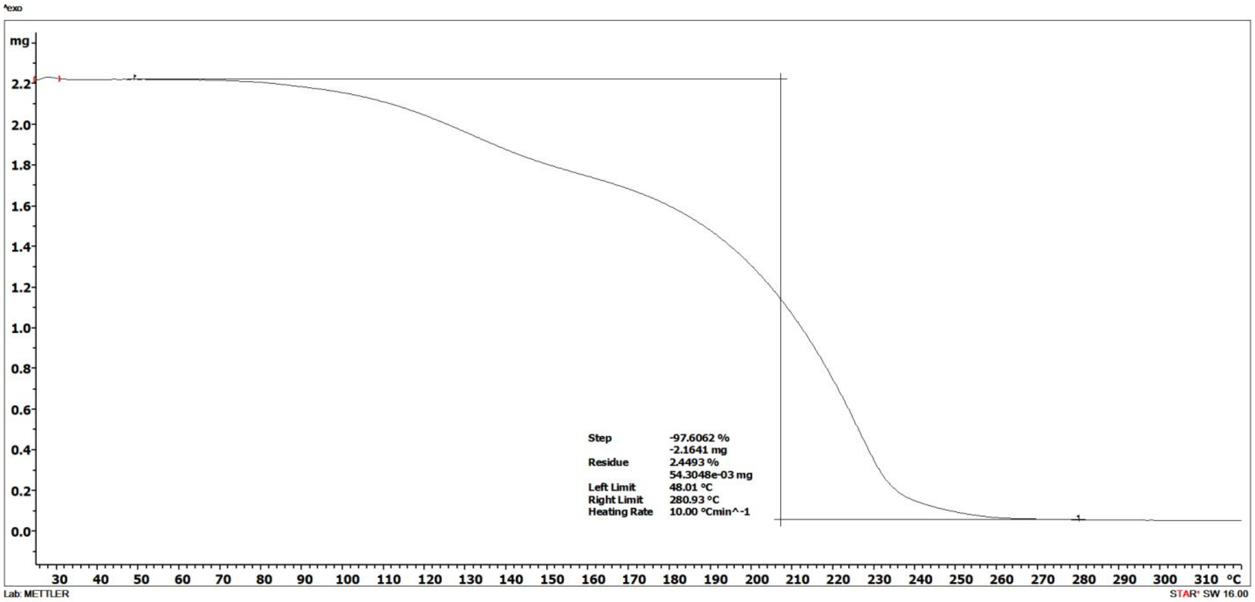

Figure S28. TGA curve of (**2**)(**13tfib**).

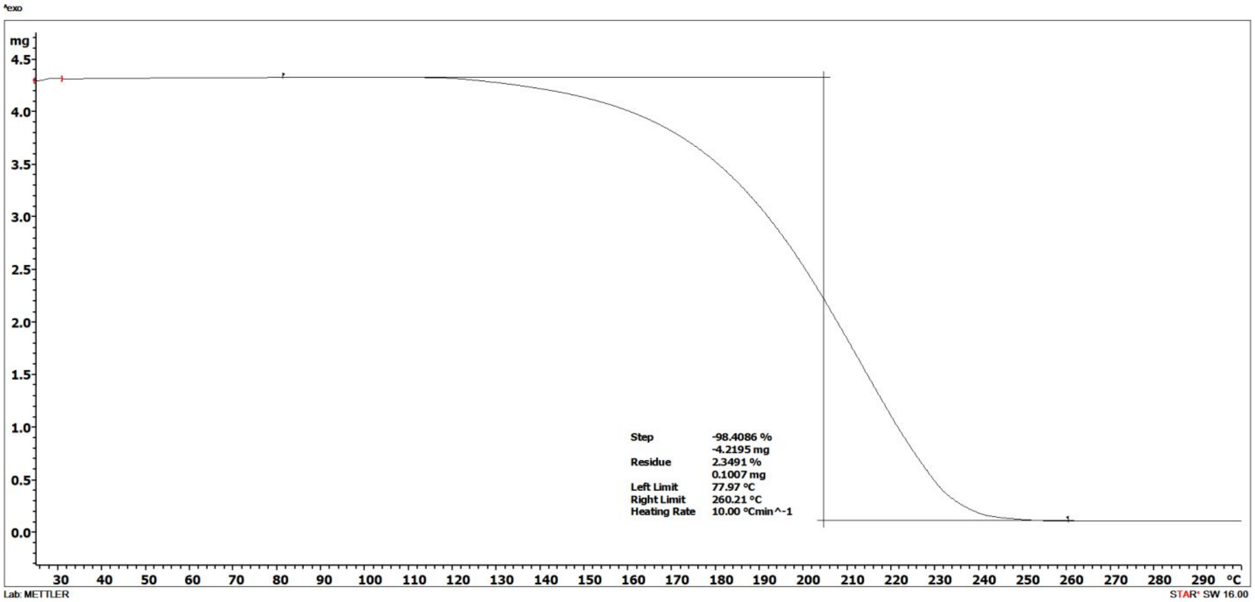

Figure S29. TGA curve of (**2**)(**135tfib**).

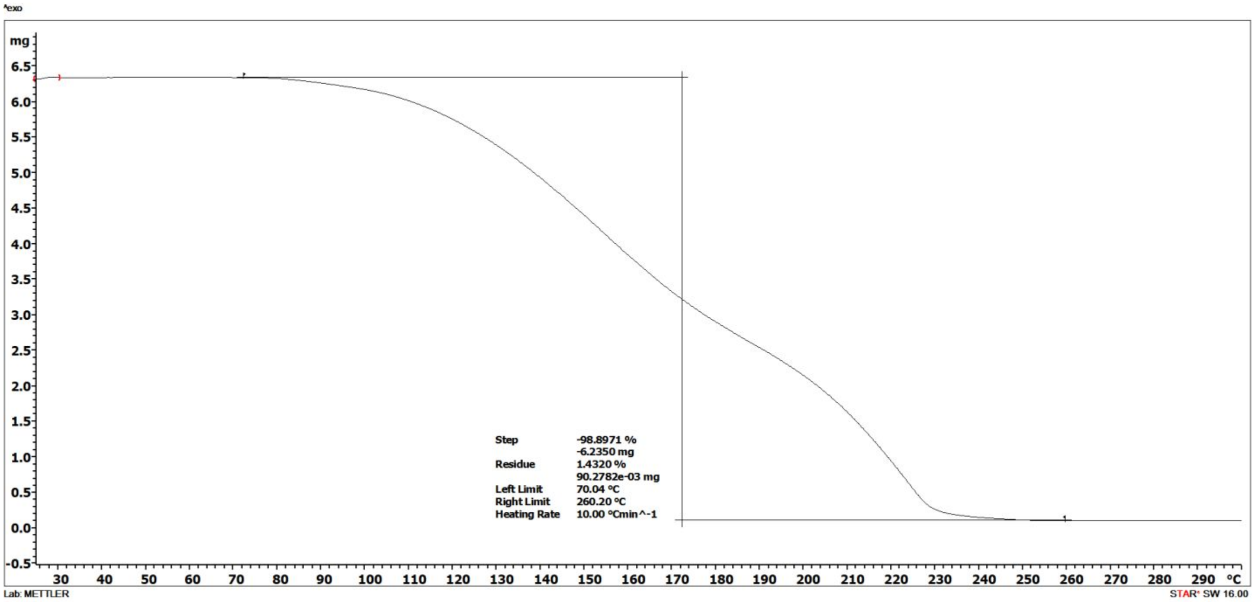

Figure S30. TGA curve of (**3**)(**13tfib**).

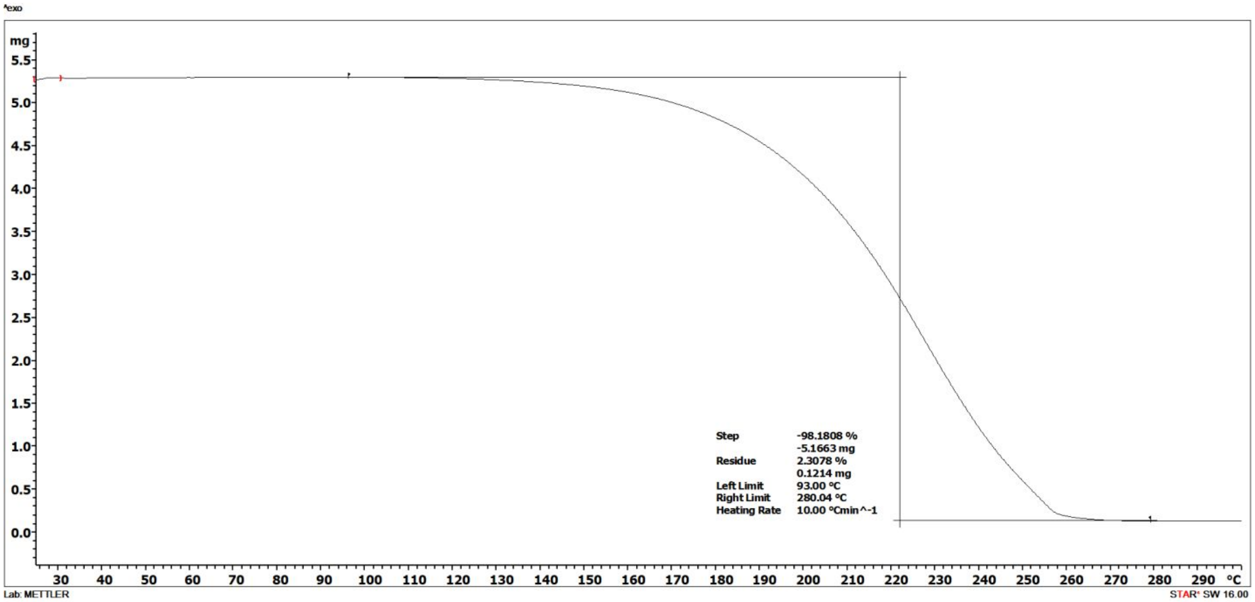

Figure S31. TGA curve of (**3**)(**135tfib**).

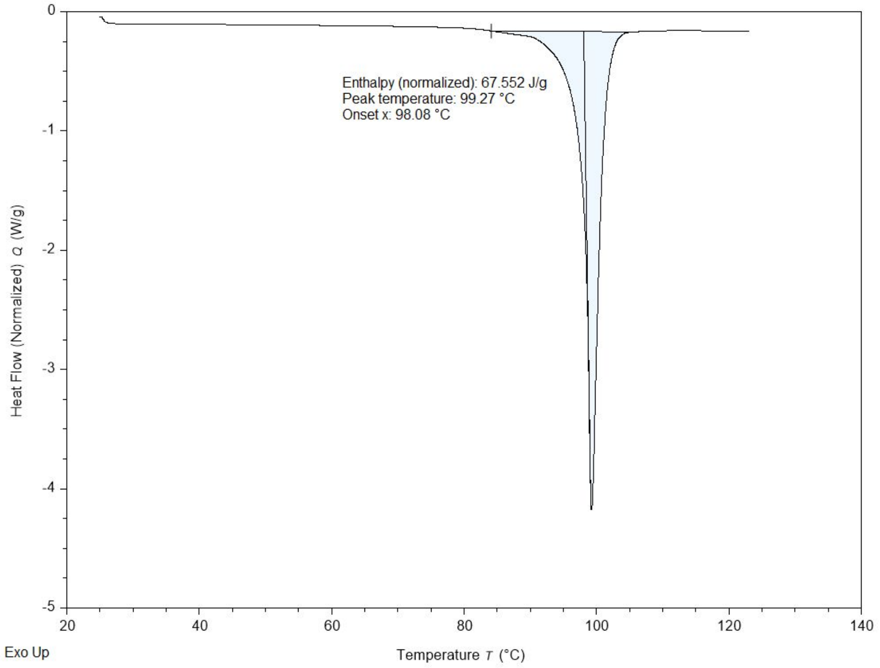

Figure S32. DSC curve of **1**.

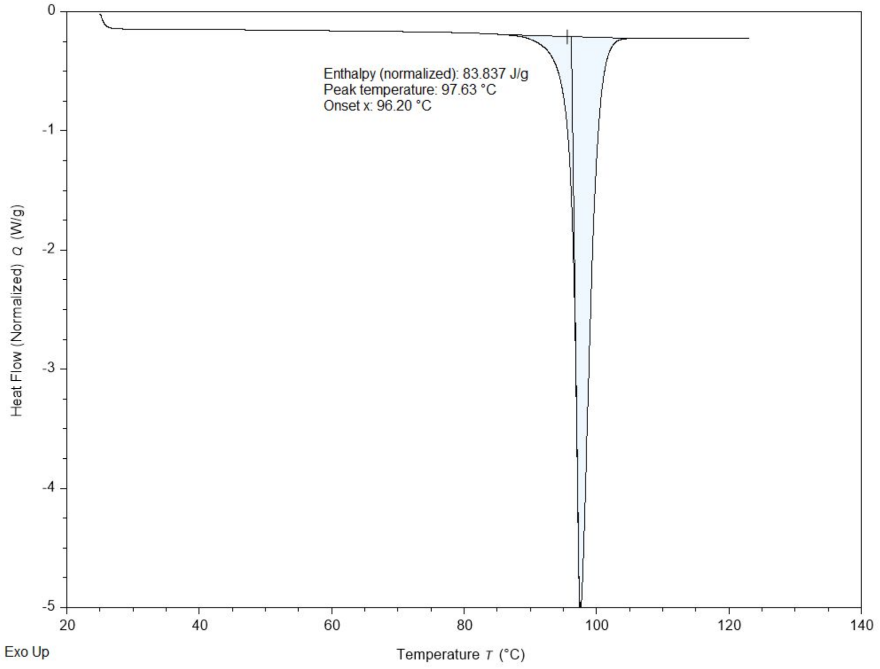

Figure S33. DSC curve of **2**.

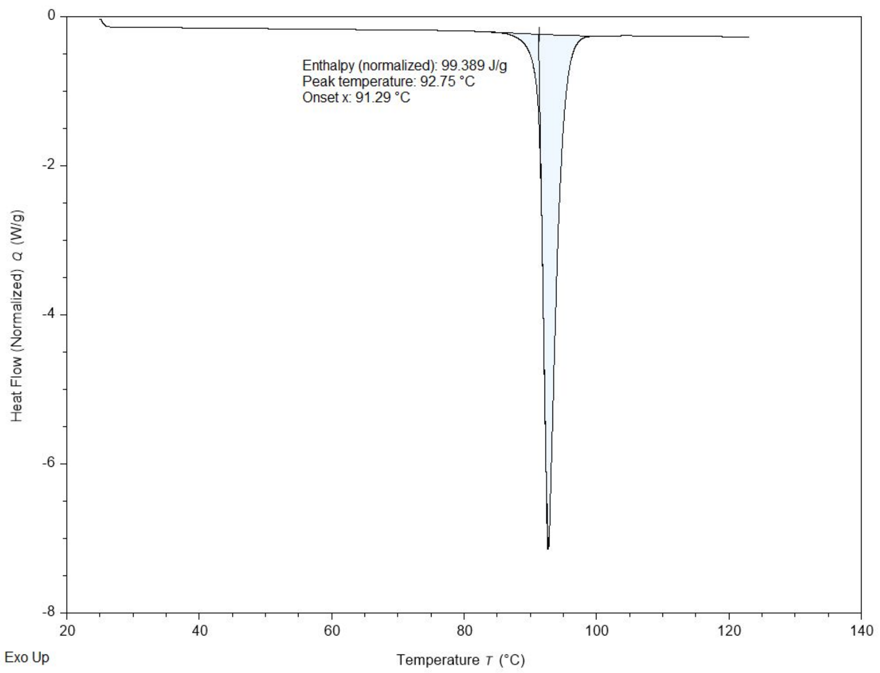

Figure S34. DSC curve of **3**.

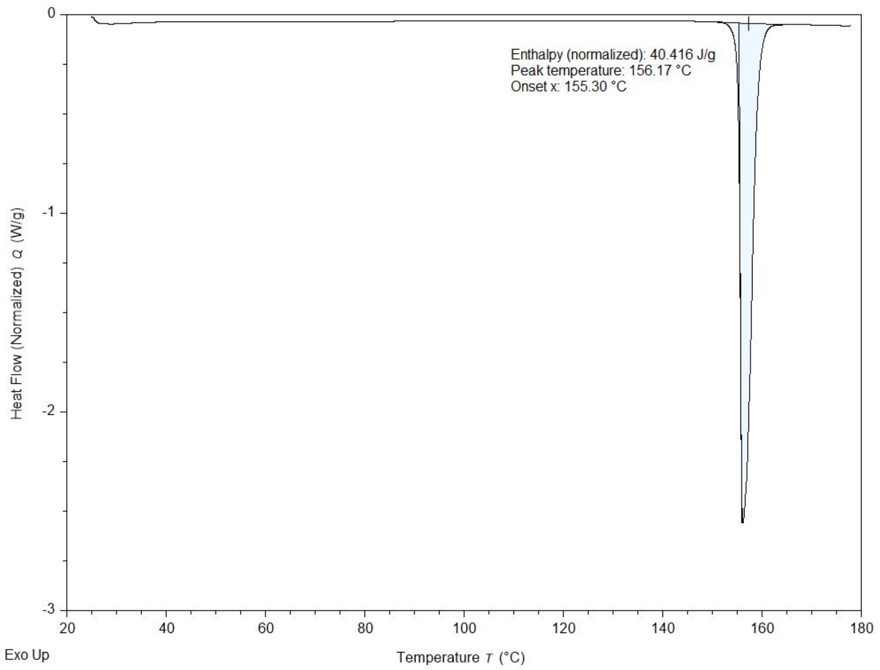

Figure S35. DSC curve of **135tfib**.

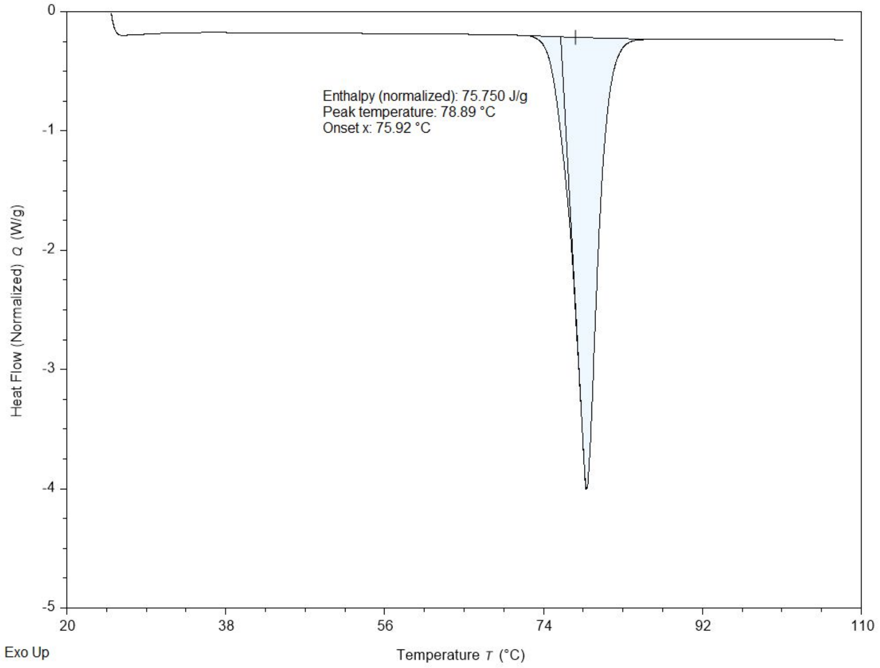

Figure S36. DSC curve of (**1**)(**13tfib**).

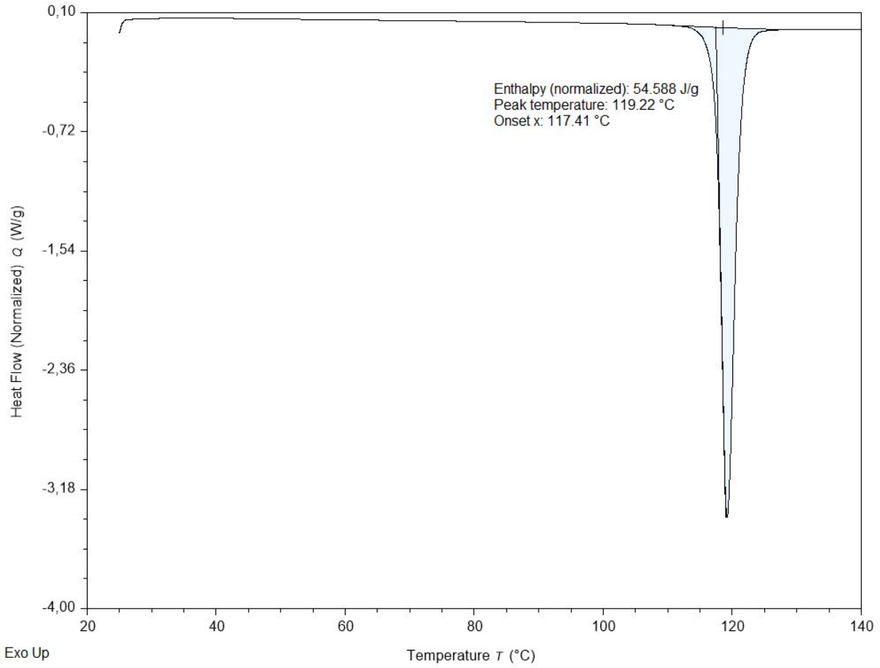

Figure S37. DSC curve of (**1**)(**135tfib**).

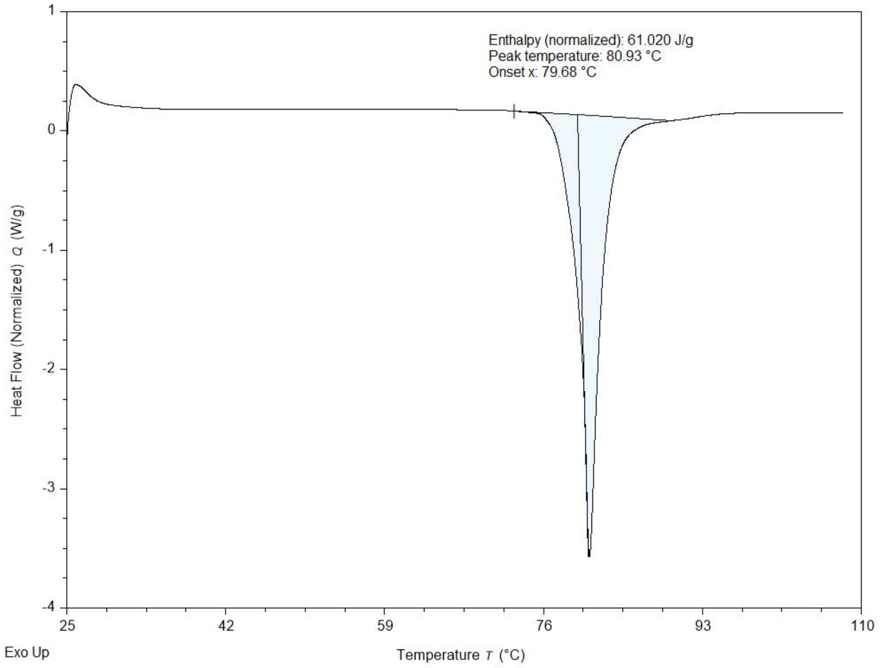

Figure S38. DSC curve of (**2**)(**13tfib**).

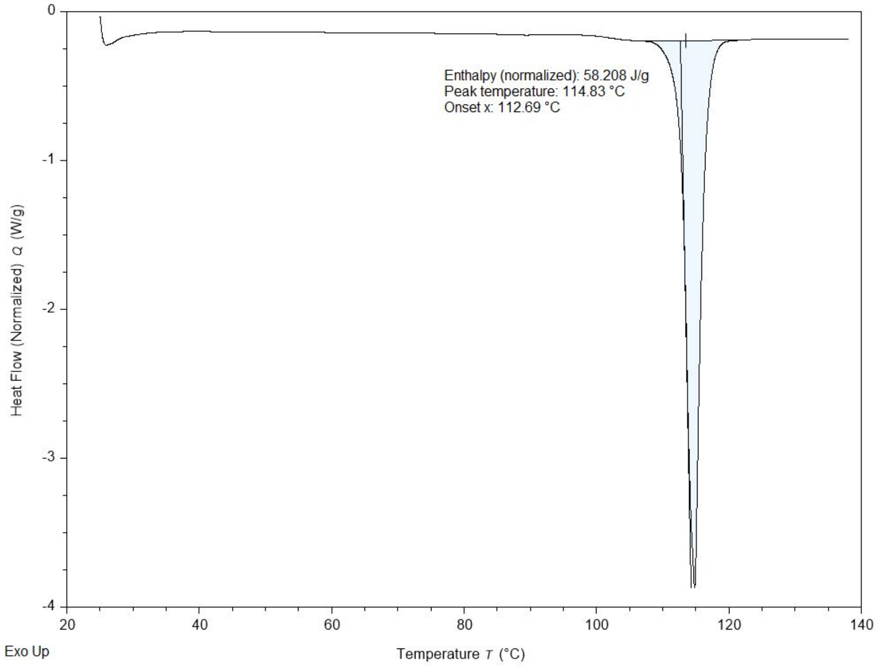

Figure S39. DSC curve of (**2**)(**135tfib**).

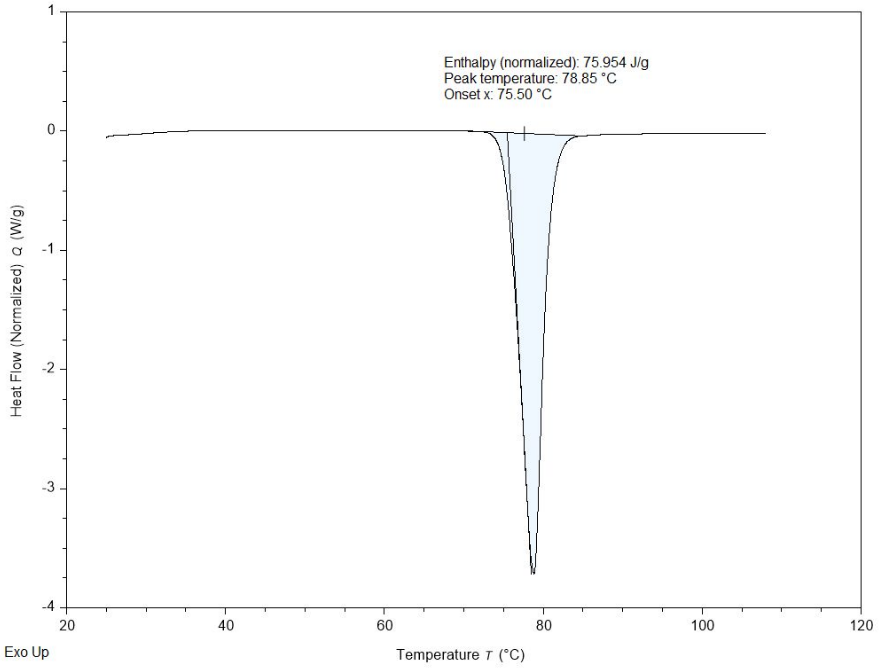

Figure S40. DSC curve of (**3**)(**13tfib**).

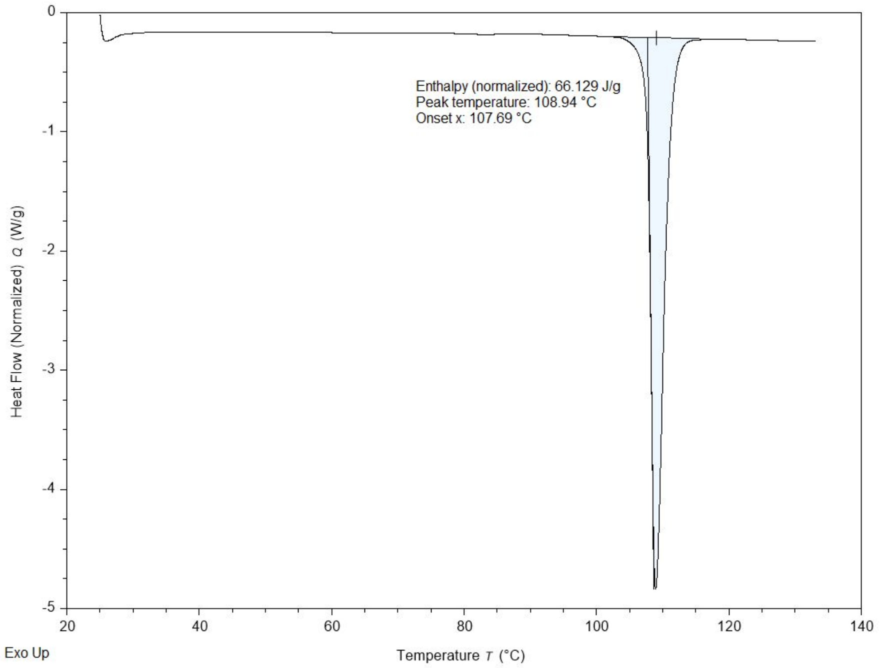

Figure S41. DSC curve of (**3**)(**135tfib**).

Table S3. Energy contributions (electrostatic E_{ele}, polarisation E_{pol}, dispersive E_{dis}, repulsive E_{rep} and total energies E_{tot}) for interactions between pnictogen-bonded imine molecules and halogen bonded imine-donor pairs in the six imine-donor cocrystals.

	O_2N□□□ONO pnictogen bond					I□□□N_{imine} halogen bond				
cocrystal	E_{ele}	E_{pol}	E_{dis}	E_{rep}	**E_{tot}**	E_{ele}	E_{pol}	E_{dis}	E_{rep}	**E_{tot}**
(1)(13tfib)	-10.9	-3.9	-19.6	9	**-24.1**	-35.3	-5.2	-21.9	48.9	**-19.4**
(1)(135tfib)	-10.3	-3.8	-19.3	8.3	**-23.6**	-36.3	-5.1	-22.8	50.9	**-19.6**
(2)(13tfib)	-12.5	-4	-19.7	9.6	**-25.4**	-36.4	-5.4	-21.5	49.7	**-19.7**
(2)(135tfib)	-12.5	-4.2	-19.3	9.6	**-25.1**	-36.3	-5.1	-22.6	50.5	**-19.7**
(3)(13tfib)	-11.7	-4	-18.5	9.1	**-23.9**	-34.2	-4.9	-21.5	45.7	**-20.4**
(3)(135tfib)	-14.9	-4	-19.6	9.6	**-27.7**	-36	-5.1	-22.1	49.3	**-19.9**

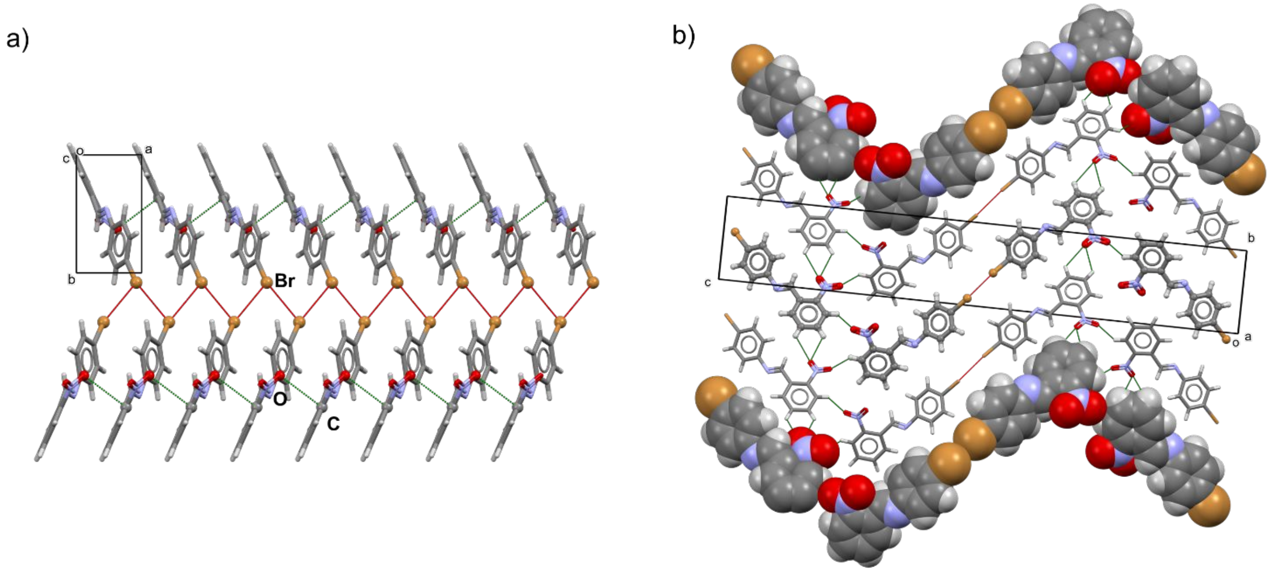

Figure S42. a) Supramolecular chains along the *a* axis in **2** achieved through Br···Br interhalogen contacts. b) interconnection of chains via C–H···O_{nitro} hydrogen bonds into 3D network in **2** viewed along the *a* axis. Interhalogen interactions are shown as red dotted lines, while the other interactions are shown as green dotted lines.

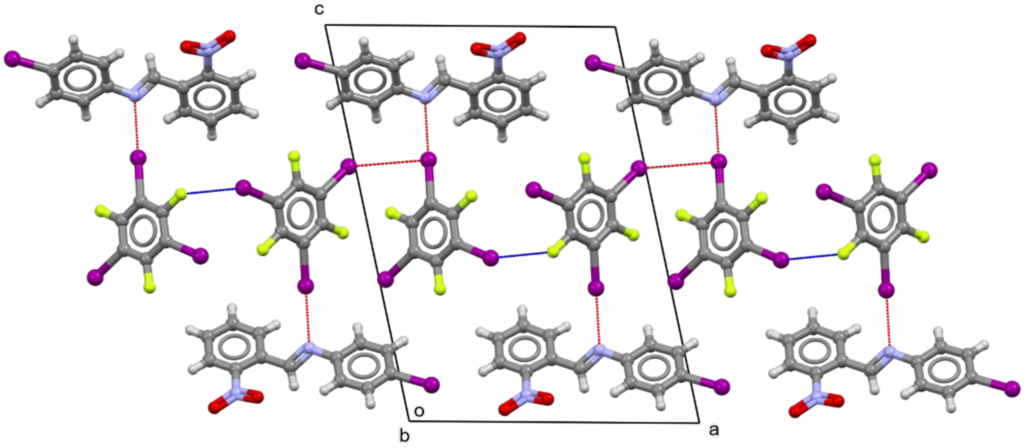

Figure S43. Interconnection of halogen-bonded chains via I···F interactions into 3D network in (**1**)(**135tfib**) viewed along the *b* axis. Halogen bonds are shown as red dotted lines, while the I···F interactions are shown as blue dotted lines.

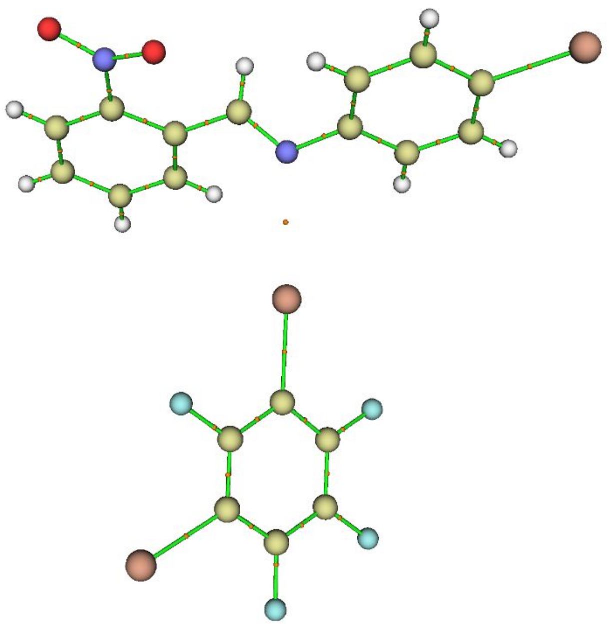

Figure S44. Bond critical points in halogen-bonded donor···acceptor supramolecular complex from cocrystal (**1**)(**13tfib**).

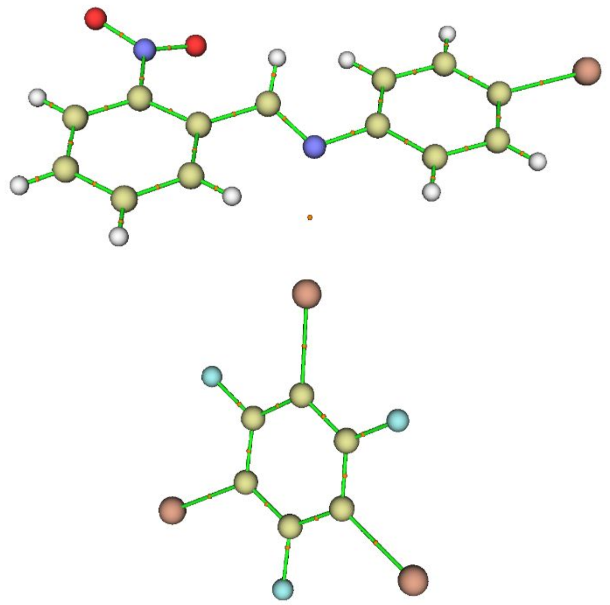

Figure S45. Bond critical points in halogen-bonded donor···acceptor supramolecular complex from cocrystal (**1**)(**135tfib**).

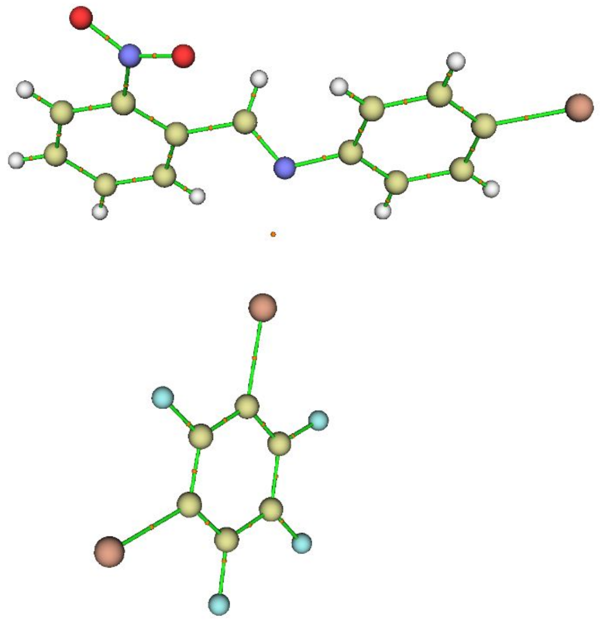

Figure S46. Bond critical points in halogen-bonded donor···acceptor supramolecular complex from cocrystal (**2**)(**13tfib**)

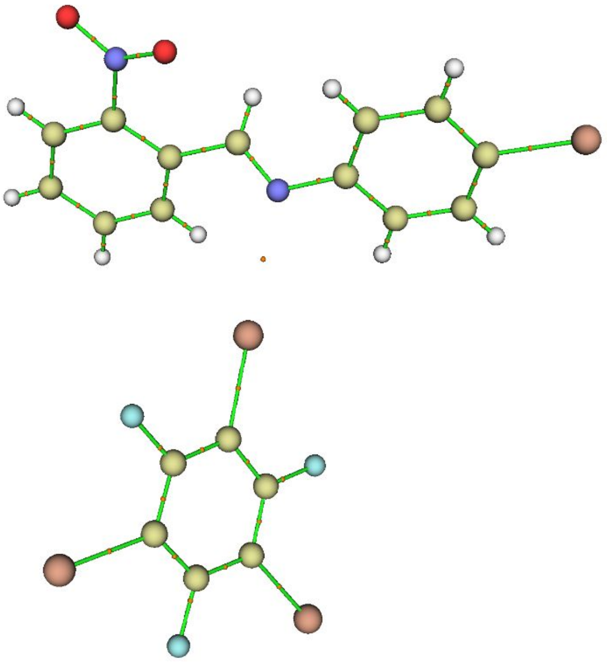

Figure S47. Bond critical points in halogen-bonded donor···acceptor supramolecular complex from cocrystal (**2**)(**135tfib**).

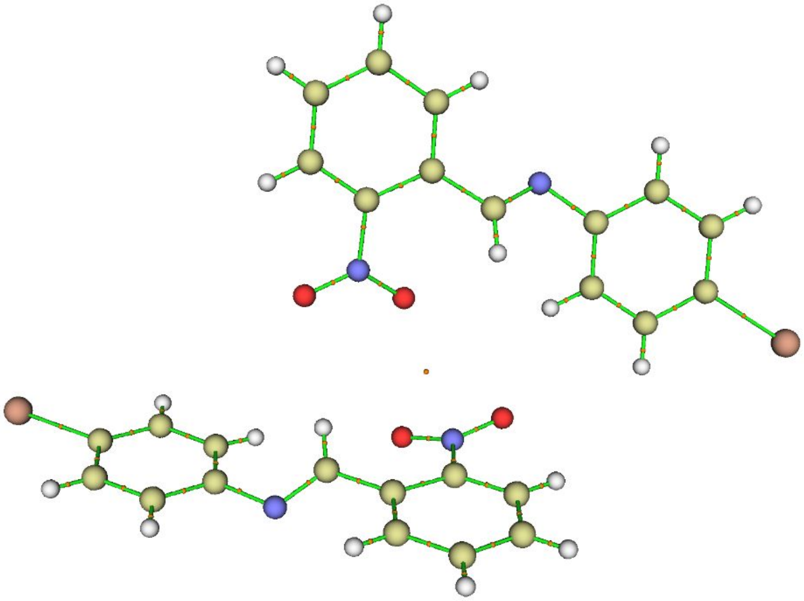

Figure S48. Bond critical points in pnictogen-bonded donor···acceptor supramolecular complex from cocrystal (**1**)(**13tfib**).

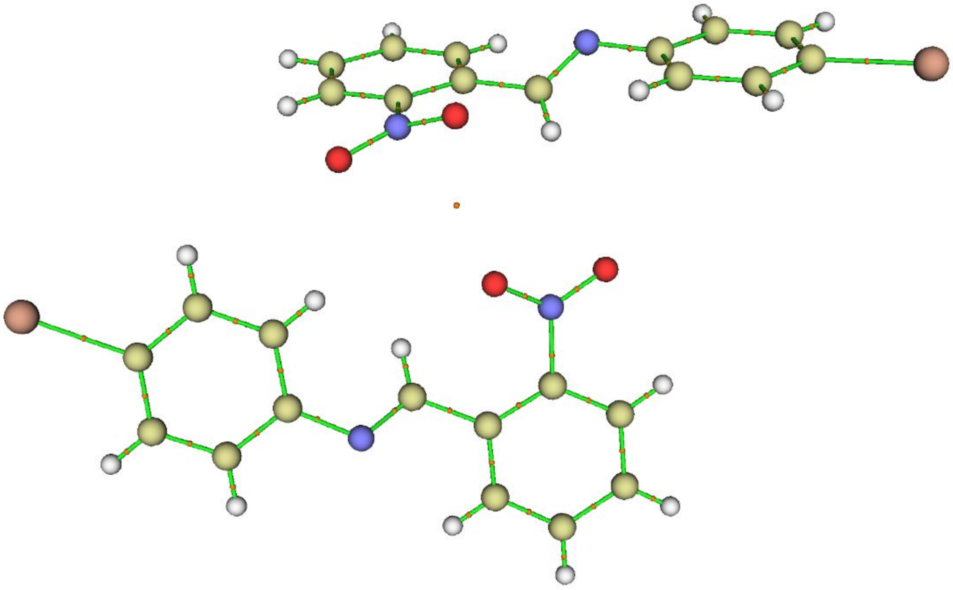

Figure S49. Bond critical points in pnictogen-bonded donor···acceptor supramolecular complex from cocrystal (**1**)(**135tfib**).

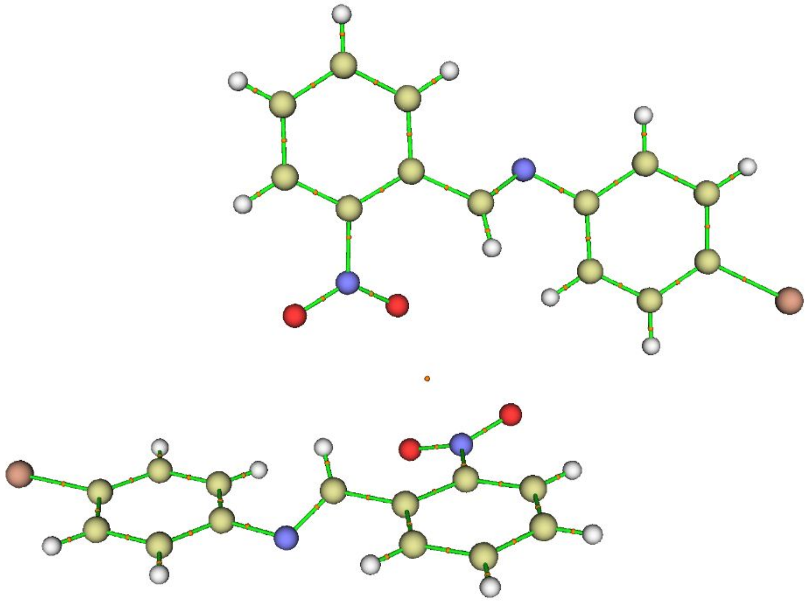

Figure S50. Bond critical points in pnictogen-bonded donor···acceptor supramolecular complex from cocrystal (**2**)(**13tfib**).

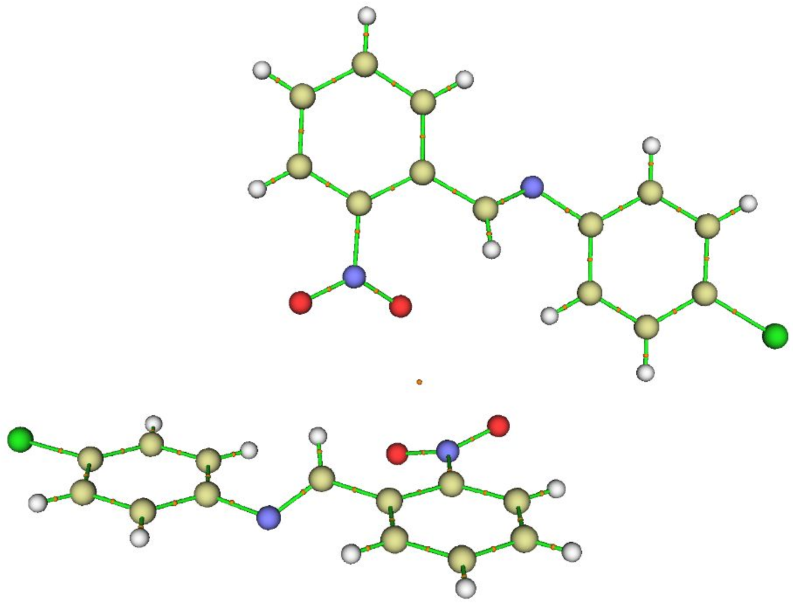

Figure S51. Bond critical points in pnictogen-bonded donor···acceptor supramolecular complex from cocrystal (**2**)(**135tfib**).

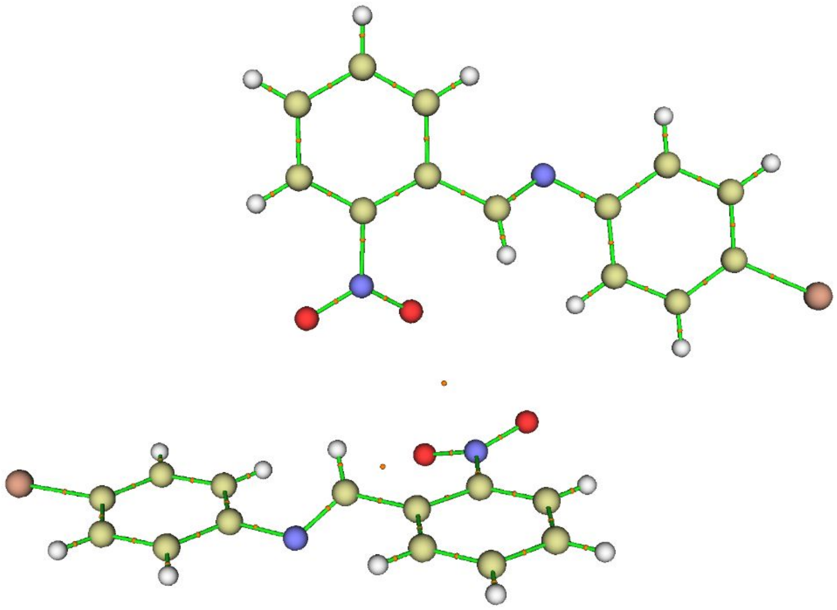

Figure S52. Bond critical points in pnictogen-bonded donor···acceptor supramolecular complex from cocrystal (**3**)(**13tfib**).

Present Addresses

† Department of Food Science, University of Copenhagen, Rolighedsvej 26, DK-1958 Frederiksberg, Denmark.
